# Kinetics of Polyampholyte Dimerization: Influence of Charge Sequences

**DOI:** 10.3390/polym16202928

**Published:** 2024-10-18

**Authors:** Seowon Kim, Nam-Kyung Lee, Youngkyun Jung, Albert Johner

**Affiliations:** 1 Department of Physics and Astronomy, Sejong University, Seoul 05006, Republic of Korea; 2 Supercomputing Center, Korea Institute of Science and Technology Information, Daejeon 34141, Republic of Korea; yjung@kisti.re.kr; 3 Institut Charles Sadron CNRS-Unistra, 6 rue Boussingault, 67083 Strasbourg, CEDEX, France

**Keywords:** polyelectrolytes, polyampholytes, dimerization, IDP

## Abstract

Polyampholytes (PAs) exhibit complex behaviors in various environments influenced by their charge distribution. This study focuses on the kinetics of dimerization of PAs, aiming to elucidate the underlying mechanisms and clarify relevant characteristics of the charge sequence. We focus on PAs with non-zero net charges, employing molecular dynamics simulations and theoretical analyses to examine how charge sequences influence the rates of dimer formation and dissociation. Our findings reveal that the charge sequence of tails and the blockiness of the minority charge group markedly influence the kinetics of dimerization: large blockiness and tails with a high number of majority-type charges slow down the dissociation of dimers. Additionally, the presence of an extended (central) block of the majority charge promotes structural diversity. Within dimer states, blocks alternate between intra- and inter-chain contacts. The duration times in the dimer states are significantly longer than the typical dwell times of block inter-contacts, with a notable extension when multiple blocks are engaged. Intrinsically disordered proteins (IDPs) play crucial roles in cellular functions, primarily due to their ability to undergo rapid conformational changes and form transient complexes. These properties largely depend on the sequence of charged residues. We provide insights into the fundamental principles governing the structural and dynamical properties of polyampholytic IDP, emphasizing the importance of sequence-specific effects on both aggregation and dissociation.

## 1. Introduction

Polyampholytes(PAs) are polymers containing both positively and negatively charged monomer units, exhibiting complex behavior in various environments influenced by their charge distribution [1,2,3,4]. A phase separation phenomenon where a homogeneous solution of polymers separates into a polymer-rich phase (coacervate) and a polymer-poor phase is a process of significant interest in materials science, biology, and pharmaceuticals. Among the various types of polymers, PAs exhibit unique coacervation behaviors influenced by their intrinsic charge heterogeneity [5,6,7,8,9]. PAs can form dense droplets through electrostatic interactions between oppositely charged segments. The stages of this process, such as nucleation, growth, and maturation are crucial in shaping the final structure and properties of the coacervates, and factors like polymer concentration, ionic strength, pH, and temperature can significantly influence it [10,11,12]. However, the pathways that govern aggregate formation are less understood, even though they play an important role in determining the effective stability, shape, and function of the aggregates.

This complexity is also observed in intrinsically disordered proteins (IDPs), which are central to numerous cellular functions, largely due to their ability to undergo rapid conformational changes and form transient complexes [13,14,15,16]. Unlike traditional proteins, IDPs do not possess a fixed or stable three-dimensional structure under physiological conditions; instead, they exist as flexible and dynamic ensembles of conformations. Their high proportion of polar and charged amino acids, along with a low proportion of hydrophobic residues, prevents them from folding into stable structures, enabling them to interact with a variety of proteins, nucleic acids, and small molecules. It has been demonstrated that charge sequence affects the conformations of overall neutral IDPs [17], stimuli responsiveness [18] and phase behavior [16,19,20]. Recent studies [21] underscore the impact of charge patterns on the structural and dynamic characteristics of liquid-liquid phase separation in IDPs. A critical aspect of polyampholitic IDP behavior is the dimerization of polyampholytes (PAs), significantly influenced by the sequence and distribution of these charged residues [22].

In our previous work, we demonstrated that the homogeneous solution of PAs can be stabilized at moderate concentrations for PAs carrying non-vanishing net charges [22]. The onset of aggregation is marked by the dimerization of identical PAs, with net charges and solvent quality serving as primary control parameters. When the net charge exceeds a critical value, charged droplets split due to an imbalance between cohesive surface energy [3] and repulsive electrostatics, a behavior reminiscent of the Rayleigh instability [23]. This phenomenon is analogous to the dimer-to-unimer transition in PAs, where increasing net charges reduce the aggregation number. In the regime where the net charge of the aggregates approaches Rayleigh instability, the detailed charge sequence of PAs becomes particularly significant [22], influencing the propensity of dimers relative to unimer states and revealing strong correlations with the blockiness of minority charges.

A key aspect of the present research is the influence of the charge sequence of PA on the kinetics of dimerization. Variations in charge distribution can lead to significant differences in the aggregation kinetics. By investigating the differences in dimerization and dissociation processes based on charge sequences, we aim to uncover the fundamental principles that govern sequence-dependent clustering kinetics. By utilizing molecular dynamics simulations and theoretical modeling for PAs, we seek to unravel the temporal evolution of aggregate structures and the factors that control their kinetics. Understanding these aspects will provide insights into designing and tailoring coacervate materials with desired properties for specific applications.

In our simulation study, we focus on PA chains whose dimer charges approach the threshold of Rayleigh instability. We identified three key factors that significantly influence the behavior and stability of charged polymer sequences:

- Block Index of Minority Charge: The longest and the second longest blocks of minority type charges directly correlate with the switching frequency, reflecting the sequence’s blockiness.
- Central Net Charge (CNC): CNC is the net charge after excluding the number of majority-type charges allocated at the first block of both chain ends. Within sequences of similar blockiness, CNC reliably predicts dimer propensity and duration, indicating the significant role of CNC in dimer stability and formation rates.
- Majority blocks: Large majority charge blocks enable the formation of loops or bridges, leading to extended dimer morphologies like the pearl necklace shape [24,25,26], which enhances structural diversity and stability.

## 2. Model and Simulation Description

We study PA chains with random charge sequences under weakly poor solvent conditions. Each PA is modeled as a bead-spring chain consisting of *N* = 100 monomers, each with a diameter σ (approximately equal to the average bond length *b*). In our model, charges are located on both end monomers and every third monomer, resulting in 34 charged monomers. Charge sequences are generated using Markovian processes, with charge sites carrying ±1 elementary charge with unbiased statistics, ensuring a global ensemble average of 〈Q〉=0. We obtained the specific *Q*-ensemble by selecting sequences with the prescribed net charge *Q* from a pool of randomly generated sequences.

**Figure 1:**
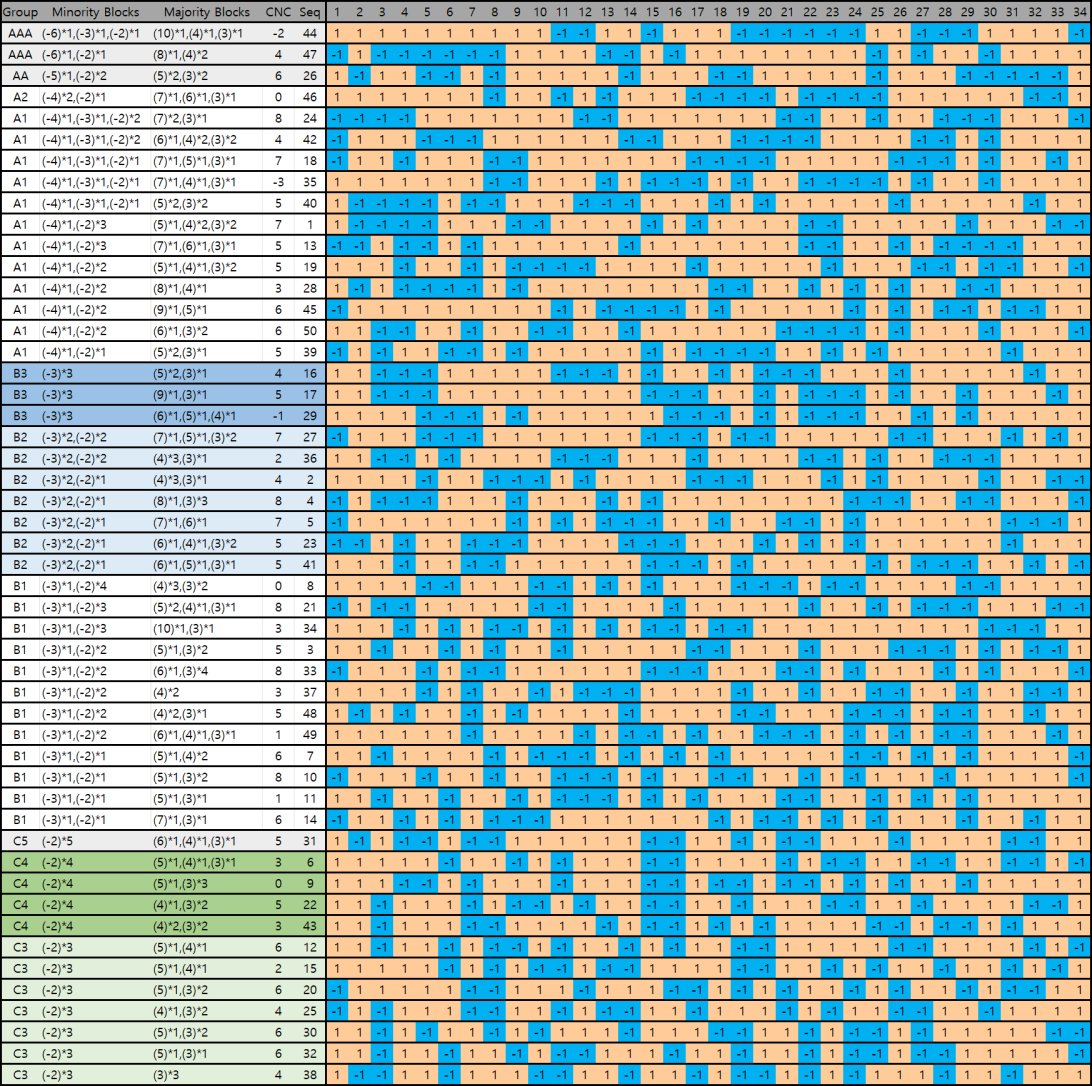
Fifty PA sequences with *Q* = 8 are organized into groups based on the blockiness of the minority charge type. Each sequence consists of 100 monomers, with 34 charged units: minority charges are represented in blue, while majority charges are in orange. For clarity, the majority and minority charge types are denoted by + and −, respectively. The lengths of the charge blocks, including all minority blocks and majority-type blocks longer than two units, as well as the corresponding CNC (Central Net Charges) values, are indicated.

In the molecular dynamics (MD) simulations, dimers are composed of 200 monomers, with 68 being charged. In a theta solvent, the critical charge for splitting a dimer into unimers is approximately $Q^{c}∼8–9$, suggesting about 4–5 units of net charge per unimer. Under weakly poor solvent conditions, the critical charge for splitting is somewhat larger; simulations indicate 8 units of net charge per unimer [22]. Hence, we sampled 50 independent charge sequences with a net charge of *Q* = 8 to investigate unimer–dimer transitions (Figure 1).

In simulations involving two chains, a unimer concentration of $2/L^{3}$ where L=30σ was used. This corresponds to a monomer concentration of $c_{0}=7.4\times 10^{-3}\sigma^{-3}$. Under the net charge and solvent conditions we considered, both the unimer state and the dimer state coexist at measurable concentrations.

The two-chain states evolve over time through unimer association and dimer dissociation (see Figure S1 for representative snapshots of these processes).

The criterion for identifying the dimer state is that the center-to-center distance between two unimers is less than 10σ (this distance corresponds to the local maximum in the free energy profile). This criterion is consistent with an alternative approach, which defines the dimer state as requiring more than four monomers to be in contact with the other chain, within a distance of 1.8σ.

All simulations are performed with implicit solvent, and solvent quality is adjusted by varying the strength of the monomer–monomer interactions, modeled using the Lennard–Jones (LJ) potential: $U_{\mathrm{LJ}}\left(r_{ij}\right)=4\epsilon_{\mathrm{LJ}}\left[{\left(\sigma /r_{ij}\right)}^{12}-{\left(\sigma /r_{ij}\right)}^{6}-{\left(\sigma /r_{c}\right)}^{12}+{\left(\sigma /r_{c}\right)}^{6}\right]$ for $r_{ij}<r_{c}$ and 0 elsewhere. Here, $\epsilon_{\mathrm{LJ}}$ and σ represent the strength and range of the LJ potential, respectively, and $r_{ij}$ denotes the center-to-center distance between particles *i* and *j*. The cutoff distance for monomer–monomer interactions is set to $r_{c}=2.5\sigma$. The theta condition in our model corresponds to a LJ parameter $\epsilon_{\mathrm{LJ}}=0.32$. We used $\epsilon_{\mathrm{LJ}}=0.60$ to represent moderately poor solvent conditions. The chain connectivity is ensured by the finite extension nonlinear elastic (FENE) potential, $U_{\mathrm{FENE}}$, between two consecutive beads [22,26].

Charged particles also interact through the Coulomb potential: $U_{C}\left(r_{ij}\right)=z_{i}z_{j}\frac{l_{B}}{r_{ij}}k_{B}T$, where $z_{i}$ and $z_{j}$ are the charge valence of particle *i* and *j*. The strength of electrostatic interactions is determined by the Bjerrum length $l_{B}$ and we set $l_{B}=3.0\sigma$. Long-range electrostatic interactions are calculated using the particle-particle-particle-mesh (PPPM) method implemented in the LAMMPS software package (https://www.lammps.org/). All dimerization simulations were conducted with two unimers of identical charge sequences. Counterions are explicitly included to ensure global charge neutrality, and excluded volume interactions for monomer-counterion and counterion-counterion are also modeled using the LJ potential. We set the interaction parameter $\epsilon_{\mathrm{LJ}}=1.0$ and the cut off distance $2^{1/6}\sigma$, leading to purely repulsive interactions.

The motion of beads is described by the Langevin equation with the total energy $U=U_{\mathrm{LJ}}+U_{C}+U_{\mathrm{FENE}}$,

$$m\frac{dv_{i}\left(t\right)}{dt}=-\zeta \frac{\partial r_{i}}{\partial t}-\frac{dU}{dr_{i}}+f^{R}\left(t\right),$$

where ζ is the frictional coefficient and *m* is the mass of the bead. Here, $r_{i}$ and $v_{i}$ are the position and the velocity of particle *i*, respectively. The Gaussian random force $f^{R}$ has zero average $\langle f^{R}\left(t\right)\rangle =0$ and correlations $\langle f^{R}\left(t\right)\cdot f^{R}\left(t^{\prime }\right)\rangle =6k_{B}T\zeta \delta \left(t-t^{\prime }\right)$ set the temperature of the system. The temperature is set to $k_{B}T=1$, and we measure energies in thermal unit $k_{B}T$. Additionally, we set ζ = 1.0 $m\tau^{-1}$ where $\tau ={\left(m\sigma^{2}/k_{B}T\right)}^{1/2}$ is the characteristic time scale. All energy measurements below are given in units of $k_{B}T$. The simulation details are provided in the Supplementary Information.

## 3. Charge Sequences of PA

The isolated PAs can exist in various states [26,27]: a uni-globule state, a pearl-necklace state [24] with multiple globules connected by open strands, or completely open if the net charge is large enough. The splitting of a charged polymer globule occurs due to an imbalance between cohesive surface energy and repulsive electrostatics when the net charge exceeds a critical value. As for the structure of a single PA chain, the sequence is especially important for partially collapsed structures like pearl-necklaces [28]. It is energetically favorable to form bridges between pearls out of overcharged subsequences which link uneven pearls provided the smaller pearls carry a higher charge density [26]. Dimer to unimer transition has some analogy to the splitting into pearl necklaces.

We group sequences based on the blockiness of the minority charges for an ensemble with Q=8, (see Figure 1 and Table 1). Initially, the sequences are classified according to their longest blocks: Group A with the longest block length of 4, Group B with the longest block length of 3, and Group C with the longest block length of 2. These groups are further categorized into sub-block structures, as previously introduced in the Ref. [22].

**Figure 2:**
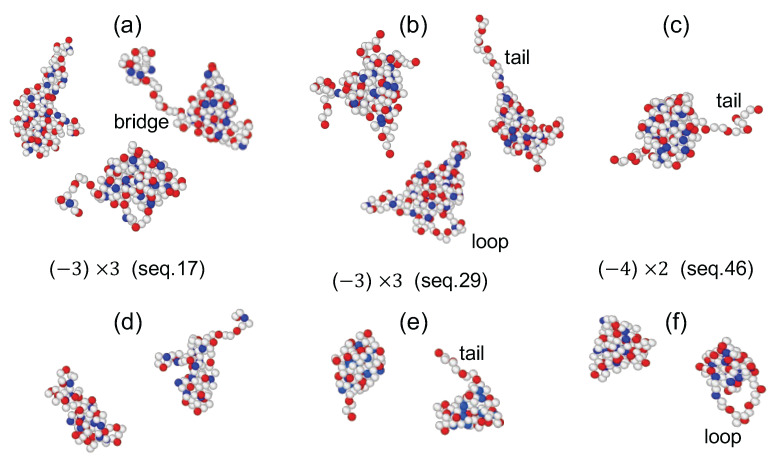
Typical dimer conformations (**a**–**c**) and a pair of unimer conformations (**d**–**f**) obtained from MD simulations for two PA chains with non-vanishing net charges of Q=8 are illustrated. Sequences 17 and 29 in (**a**,**b**,**d**,**e**) contain three blocks of triple minority charges ((−3)×3), while sequence 46 in (**c**,**f**) contains two blocks of quadruple minority charges ((−4)×2). In these visualizations, red represents majority charges (+) and blue represents minority charges (−).

We introduce a parameter called the “central net charge” (CNC) to represent the net charge of the core region by simply extracting the number of majority-type charges allocated at the first block of both chain ends. By redistributing charges, dimers can be further stabilized. For quenched charge sequences, it is possible to exclude charges on the tails from the core, forming a more compact and less charged core (see Figure 2). Hence, CNC values reflect the stability of the PA cores. The CNC value of each sequence is given in the table (see Figure 1).

## 4. Static Properties and Contact Maps

Statistical properties were previously studied [22]. Here, we provide a brief summary illustrated by quantities not presented earlier.

**Figure 3:**
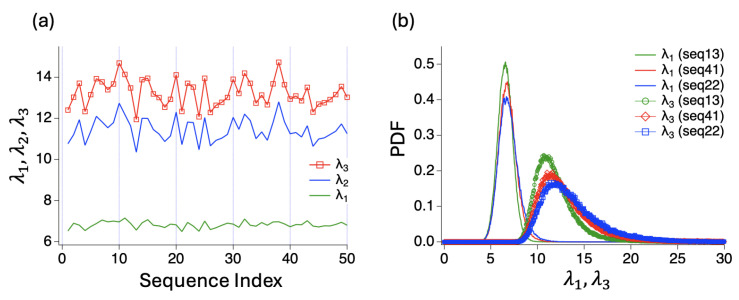
(**a**) Three eigenvalues $\lambda_{1}\leq \lambda_{2}\leq \lambda_{3}$ of inertia tensor for 50 sequences. The eigenvalues are obtained for dimer states and averaged over throughout the simulation times. (**b**) Distributions of eigenvalues $\lambda_{1}$ and $\lambda_{3}$ for three sequences (seq.13(A1), seq.41(B2), seq.22(C4)) with the CNC values of 5.

To investigate the influence of charge sequences on the shape of dimers, we measured three eigenvalues $\lambda_{1}\leq \lambda_{2}\leq \lambda_{3}$ of inertia tensor for 50 sequences and obtained the time average of each eigenvalues for dimer states. As shown in Figure 3, $\lambda_{1}<\lambda_{2}\approx \lambda_{3}$. Table S1 in Supplementary summarizes the eigenvalues for the conformations depicted in Figure 2. Note, that elongated shapes are characterized by two large eigenvalues. We also present the distributions of eigenvalues $\lambda_{1}$ and $\lambda_{3}$ for three representative sequences—13(A1), 41(B2), 22(C4)—all with the CNC values of 5. The peaks of these distributions shift to larger values in the order seq.13 < seq.41 < seq.22, indicating that more blocky sequences are more compact.

**Figure 4:**
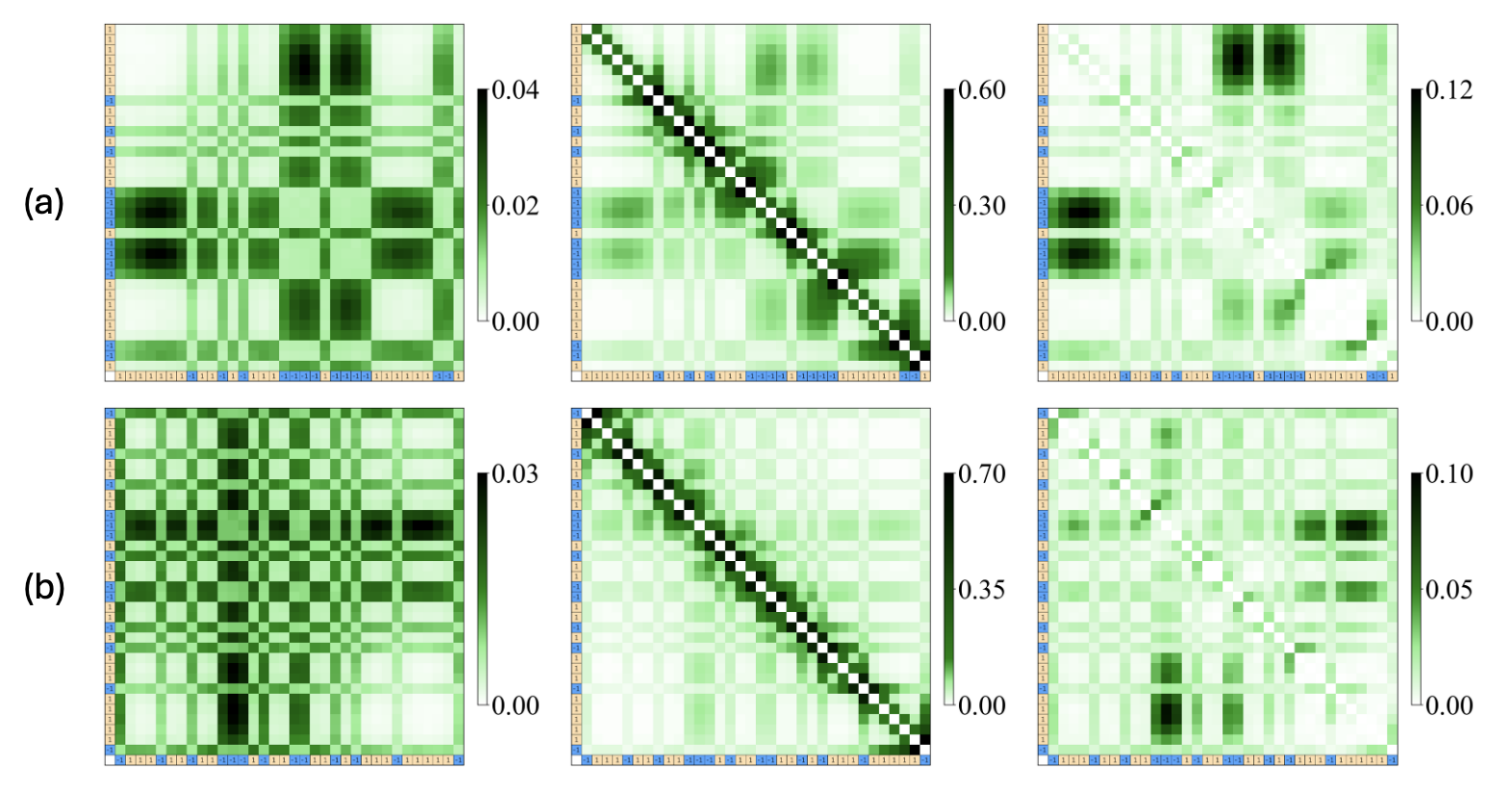
Contact maps for representative sequences: (**a**) seq.46 (A2) and (**b**) seq.10 (B1). The charge sequence is shown in top rows and most left column, where blue and orange colors indicate minority and majority charge types, respectively. The intensity of the green color represents the contact frequencies, as indicated by the scale bar. The left panels display inter-chain contact maps, while the middle panels show intra-chain contact maps in the dimer states The right panels depict the difference in the intra-chain contact probability between the dimer state and the unimer state.

The contact map illustrates the frequency of monomer–monomer contacts between charged monomers (Figure 4). Two monomers are considered to be in contact if the distance between them falls within 1.8 σ. The inter-chain contact maps (left panels in Figure 4) counts the contacts between monomers belonging to different chains, while the intra-chain contact maps (middle panels in Figure 4) count the contacts between monomers within the same chain. In the map, the given (identical) charge sequence is shown in the top rows and most left columns. Blue and orange colors indicate minority and majority charge types, respectively. The intensity of green colors indicates the level of contact frequencies. As depicted in the contact map, frequent contacts are primarily established by blocky minority charges, highlighting the crucial role of these blocks. The right panels of Figure 4 illustrate the differences in intra-chain contact probability between the unimer and dimer states. The most significant changes are observed in the contacts between monomer pairs within the longest minority blocks and the distant majority blocks. Upon dimer formation, less than half of the intra-chain contacts are partially replaced by inter-chain contacts, with fewer changes occurring between adjacent monomer pairs. We also observe that the two chains are not well mixed, as reflected by the overall low inter-chain contact probability (see, also the movie files in the Supplementary). The intra-chain contact probability remains higher, even for distant monomer pairs. This suggests that intra-chain interactions continue to dominate, leading to relatively limited inter-chain mixing during dimerization.

We also checked counterion condensations in unimer and dimer states [22,29]. When PAs are in a dimer state, the number of counterions located in the vicinity of dimers (within 1.8σ from the closest monomer) was consistently observed to be 4–5 (out of 16), regardless of the sequence type, with a sequence average of 4.5 ± 0.1. When PAs are in a unimer state, the number of counterions per unimer was observed to be 1.63 ± 0.03.

## 5. Dwell Times and Survival Probabilities

**Figure 5:**
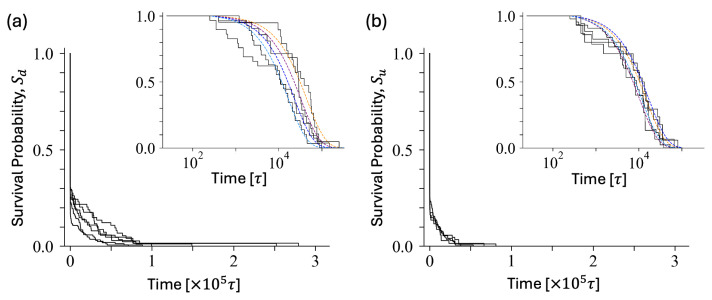
The survival probability (**a**) $S_{d}$ for dimer and (**b**) $S_{u}$ for unimer states for some representative sequences. Exponential fits in insets are displayed in different colors for each blockiness group: seq.19 (A1, yellow), seq.16 (B3, purple), seq.4 (B2, blue) and seq.13 (B1, green). For the presentation, the population is reset as 1 at time *t* = 250τ, that is 35% of the initial population for dimers and 17% for unimers.

We measured the durations of dimer and unimer states (dwell times) for each sequence. The dwell times of dimer states and unimer states, denoted by $p_{d}\left(t\right)$ and $p_{u}\left(t\right)$, capture the switching events within the time interval [t,t+dt] from dimer to unimer and vice versa. A large number of switchings occur at small times (<250τ), indicating transient states. Both the dimer and unimer populations drop quickly after the first dimerization/dissociation attempt. About 32 ± 8% of the dimer population survives beyond 250τ, while about 26 ± 7% of the unimer population survives beyond 50τ. At large times, the population decays exponentially, $∼\exp(-t/\tau_{r})$. The survival probabilities $S_{d(u)}=1-\int_{t_{c}}^{t}p_{d(u)}\left(t^{\prime }\right)dt^{\prime }/\int_{t_{c}}^{\infty }p_{d(u)}\left(t^{\prime }\right)dt^{\prime }$ for each state, with the lower cut-off $t_{c}=250\tau$ are shown in Figure 5. The characteristic times $\tau_{r}$ for the dimer state vary significantly from one sequence to another within the Group, with more pronounced variation in Group A. In contrast, unimer states show consistent characteristic times. Switching frequency from a dimer state is primarily influenced by blockiness.

**Figure 6:**
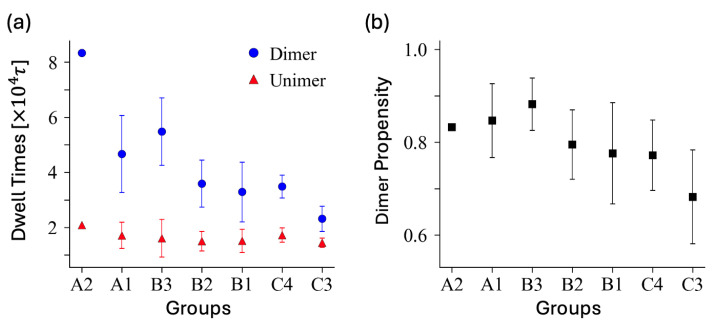
(**a**) The blockiness dependence of the average dwell times $\langle t_{d}\rangle$ for the dimer state and $\langle t_{u}\rangle$ for the unimer state considering those that survived longer than 250τ. There is anti-correlation between unimer and dimer dwell times for block sequences. (**b**) The average values of dimer propensity within each group are presented in the right panel for comparison.

The sequence average of the characteristic times from exponential fits is 4.7, 5.3, 3.7, 3.5, 3.5, and 2.5 (in units of $10^{4}\tau$) for A1, B3, B2, B1, C4, and C3, respectively. When taking the average for the matured dimers (lasting more than 250τ), we obtain 4.7, 5.5, 3.6, 3.4, 3.5, and 2.3 (in units of $10^{4}\tau$), which is consistent with the fitted times. Figure 6 shows the average dwell times of the dimer and unimer states for matured dimers and unimers, as well as their correlation with blockiness. When taking the simple average for all dimers (including short-lived ones), the values are approximately three times smaller: 1.4, 1.9, 1.2, 1.1, 1.1 and 0.7 (in units of $10^{4}\tau$).

Focusing on the more typical sequences (those with >5% occurrence), as shown in Table 1, we find the average dwell times (after 250τ) in the following order: B3 > A1 > B2 > B1 ≈ C4 > C3. This hierarchy suggests that sequences with more pronounced block structures exhibit longer dwell times. Notably, the sequence A1 has no more than one instance of the second longest block (triplet). The presence of blockiness appears to retard the switching kinetics. This observation is consistent with the data, implying that sequences with larger blocks tend to remain stable for extended periods before transitioning to other states. The stability induced by blockiness can be attributed to the increased energetic or kinetic barriers for transitions, thereby prolonging the residence time within these sequences.

**Figure 7:**
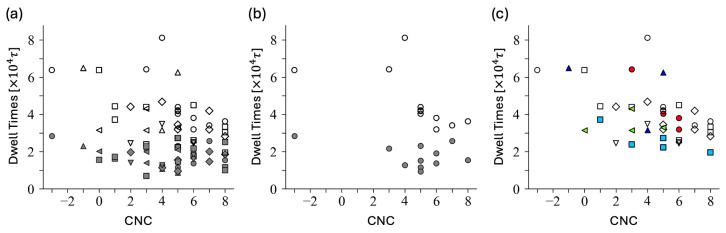
(**a**) The correlation between CNC and the average dwell times is shown for various blockiness groups in (**a**) and with a specific focus on group A1 in panel (**b**). Symbols represent different groups: A1 (◦), B3 (△), B2 (◇), B1 (☐), C4 (◁), C3 (▽). The dwell times $\langle t_{d}\rangle$ at the dimer state are represented as open symbols, while the dwell times $\langle t_{u}\rangle$ for the unimer state are shown as filled grey symbols. (**c**) The average dwell times $\langle t_{d}\rangle$ highlighted for selected subgroups: 4 sequences with (−4)×1,(−2)×1 (red ◦); 3 sequences with (−3)×3,(−2)×0 (navy △); 5 sequences with (−3)×1,(−2)×2 (light blue ☐); 4 sequences with (−2)×4 (light green ◁).

Blockiness is better defined by further considering the second longest blocks. When subgroups down to a block size of 2 are considered, CNC effectively characterizes the length of dimer dwell times (Figure 7). Within the group of sequences with equivalent blockiness, dimer dwell times generally correlate well with the CNC values, with only a few exceptions. Small CNC implies that dimer cores are likely much below the Rayleigh instability, hence dimer states are stable against fluctuation. This correlation can be demonstrated within each subgroup that has equal block sizes: four sequences of Group A1 with (−4)×1,(−2)×1 and five sequences of Group B1 with (−3)×1,(−2)×2, as shown in Figure 7c. The Pearson correlation coefficients are −0.97 and −0.84, respectively. On the other hand, four sequences with (−2)×4 (Group C) show very weak correlations. It is difficult to quantify the correlation for three sequences of Group B3 with (−3)×3,(−2)×0 due to the small sample size and the exceptional behavior of seq.17, as discussed later.

For the unimer states, the correlation of CNC and the dwell times exhibit a fair correlation for block sequences (A groups and B3) and less for B2. Correlations are not evident for less blocky sequences (B1 and C), as shown in Figure 7a,b.

In the Group A1 samples, subgroups with and without triples are mixed. As shown in Figure 7b, while dimer dwell times correlate with blockiness and CNC, the unimer dwell times remain approximately 1.8±0.6 (in units of $10^{4}\tau$). Sequence 39, the least blocky in Group A1, indeed has the shortest unimer dwell time of around 0.9 ×$10^{4}\tau$.

There are three Group B3 sequences: 16, 17, and 29. The CNC values are 4, 5, and −1, respectively. No other blocks are present besides triples. The unimer dwell times are well correlated with CNC values. Sequence 29 with the CNC value of −1 exhibits a longer unimer dwell time (factor 2 longer than the other two sequences). The dimer dwell times of both sequences 17 and 29 are similarly long (approximately $6.5\times 10^{4}\tau$), despite their very different CNC values. The long dimer dwell time for seq.17 is unexpected. We found that sequence 17 often exists in two-pearl conformations, allowing it to remain as a dimer after splitting, as shown in Figure 2a. The pearl necklace form of sequence 17 is attributed to the long majority-type block that separates the minority-type blocks, indicating the substantial role of the long majority block. Similarly, among the Group B1 ((−3)×1), seq.34 has a dimer dwell time twice as long as seq.37, despite both having the same CNC value of 3.

**Figure 8:**
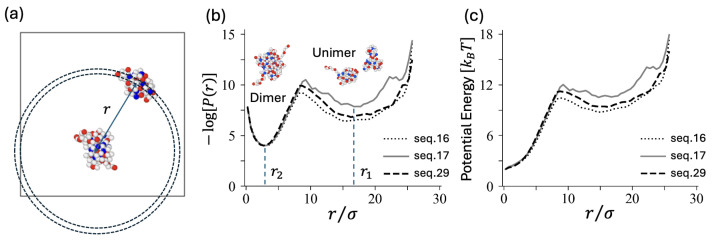
(**a**) Schematic illustration showing the reaction coordinate *r* as the center-to-center distance between unimers. (**b**) The probability distribution P(r) of finding another unimer’s center within a spherical shell defined by the range [r,r+dr] is plotted as −logP(r) with an arbitrary shift for three sequences of Group B3. The positions of the two minima, $r_{1}$ and $r_{2}$, represent the stable unimer and dimer states, respectively. (**c**) Potential profiles V(r) derived from the probability density.

In order to compare the free energy barriers associated with dimerization and dissociation for each sequence, we evaluated populations of the second unimer taking the center-to-center distances *r* as reaction coordinates (Figure 8a). The function P(r) counts the probability of finding another unimer’s center within a spherical shell defined by the range [r,r+dr]. The potential profiles V(r) are obtained as V(r)=−logP(r)+2log(r/σ), where the term 2logr accounts for the entropy associated with the spherical shell volume at a distance *r* (Figure 8c). Some potential profiles of the two PA systems under study are shown in Supplementary (See, Figure S2).

## 6. Fokker–Planck Type Approach

Below we propose an estimate of typical transition time (dwell times) in both directions. We can construct a qualitative expression involving the width of the barrier, the width of the starting sink, and the height of the barrier. Here, we consider the center-to-center distance, denoted as *r* in Figure 8, as the reaction coordinate. The associated friction may affect the entire globule or certain protruding parts that could fluctuate near the top of the barrier, approximately $k_{B}T$ below the maximum. The qualitative argument goes as follows: The system explores the starting potential sink over some shell of volume V where the free energy remains within thermal energy from the local minimum. The volume V defines the thermalized probability right under the barrier opposing diffusion towards the target sink as $\exp(-V^{*})/V$ with $V^{*}$ the potential increase up to the top of the barrier. Therefore, the flux through the shell at the top of the barrier is deduced as $D4\pi r_{*}^{2}\left(\exp\left(-V^{*}\right)/V\right)/L_{*}$, with $L_{*}$ the width $k_{B}T$ under the barrier. The characteristic time of the escape process is obtained as the inverse probability flux into the target sink. This simple estimate provides the correct order of magnitude for dissociation but not for dimerization, where the barrier is small and all details matter, as there is no effective thermalization in the starting sink.

We can tentatively describe the dimerization/dissociation process based on the sole center-to-center distance, *r*. The dimer state corresponds to the shell around the close minimum, $r_{2}$, while the dissociated state corresponds to the shell around the far minimum, $r_{1}$ (see, Figure 8). We assume spherical symmetry. The Fokker–Planck equation expresses the conservation of probability:

(1) $$\frac{\partial P}{\partial t}+\nabla .j=0$$

where j is the probability current. We assume spherical symmetry which the probability current is as follows:

(2) $$j=-D\frac{\partial P}{\partial r}-DP\frac{\partial V}{\partial r}.$$

**Figure 9:**
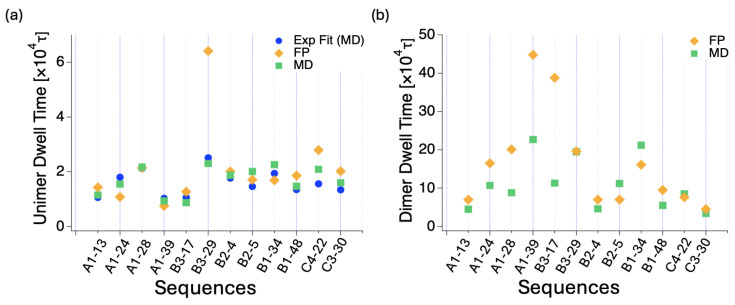
Comparison of theory (FP calculation) and simulations for sequences indicated in the *x*-axis ticks. (**a**) Dimerization times and (**b**) Dissociation times.

The FP equation can be solved numerically by incorporating free energy profiles as studied earlier, [30]. The potential *V* is linked to the probability per unit volume by taking −log and is shown in Figure 8c. We extracted sixth- to eighth-degree polynomials, which were fitted and incorporated into the FP equation. The formal diffusion constant *D* describes the fluctuation of the center-to-center distance. It is twice the diffusion constant of the center of a unimer. A compact formula for the mean first passage time between the two minima is given in the Supplementary together with more details on the FP equation including boundary conditions. An alternative approach considers the basin, rather than the strict minimum, to define a state. It is important to note that this definition of dimer and unimer states is not strictly equivalent to using a criterion based on the number of contacts. For dimerization, the FP and simulation results yield fairly similar characteristic times, except for seq.29, which is very compact with a majority-type tail (or sometimes a corona) that protects unimers from approaching and associating. Although the potential *V* captures some of the effects, the ultimate coalescence dynamical mechanism is more complex. Dissociation shows slightly more discrepancy. Sequences that fluctuate and visit pearl-necklace conformations (e.g., seq.17 and seq.39) are less well described by the spherically symmetric FP equation, as these pearl-necklace-shaped dimers provide alternative paths for dissociation. A direct comparison between FP and simulation results is shown in Figure 9.

## 7. Dynamic Contact Maps

**Figure 10:**
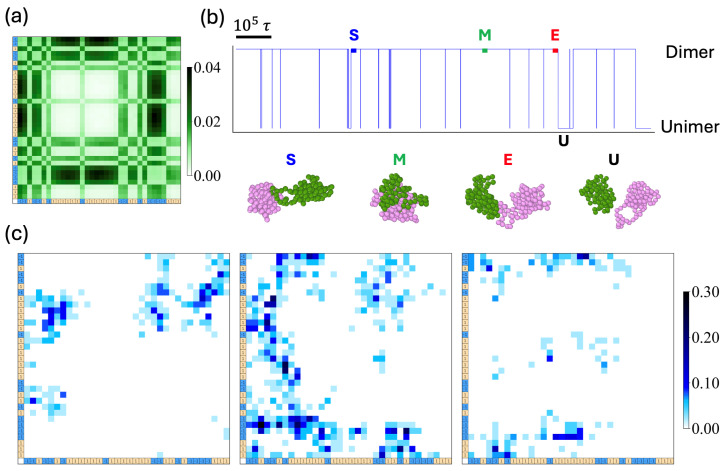
(**a**) Long time (∼$1.2\times 10^{6}\tau$) accumulated contact map and (**b**) a time trajectory of two-chain states switching between dimer states and unimer states for seq.13. The snapshots display the conformations at the beginning, middle, and end of the dimer state, labeled as S, M, and E, respectively, with the unimer state marked as U. (**c**) Three dynamic contact maps, accumulated for 250τ are obtained at the times indicated as S, M, and E. The intensity of the color bars represents contact probability on a scale from 0 to 0.3.

We introduced a dynamic contact map, in order to demonstrate the evolution of block–block correlations (Figure 10). Each contact map measures the frequency of inter-contacts between designated monomer pairs over the duration of ∼250τ.

We chose three time windows for analysis: immediately after the dimer is established (S), after the aging of the dimers (M), and shortly before exiting the dimer state (E). The contact maps clearly illustrate the significance of tail sequences, where minority blocks are linked to regions of the other chain, which are predominantly occupied by majority-type charges, at both the beginning and the end of the dimer state.

## 8. Survival Times of Inter Block Contacts

**Figure 11:**
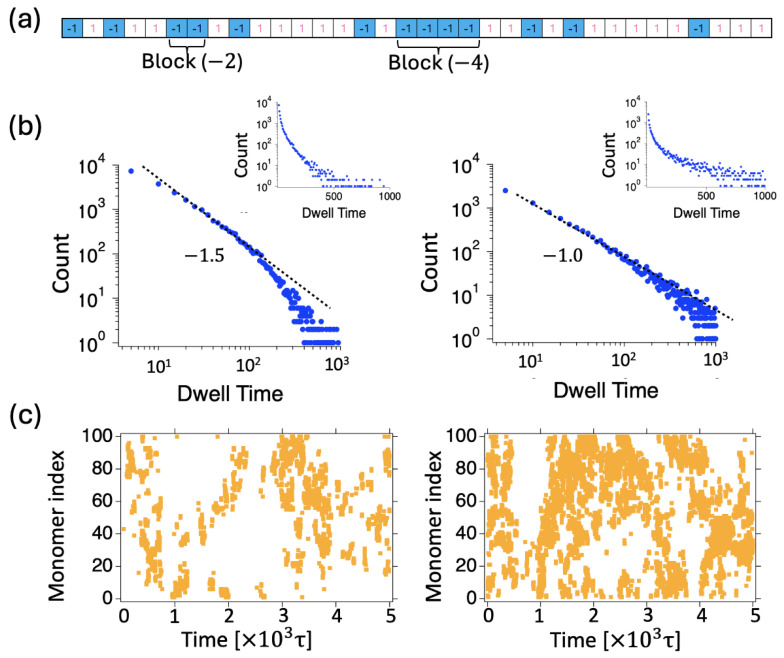
(**a**) Sequence 39 with two tagged minority charge blocks: a Block of (−2) and a Block of (−4). (**b**) The intercontact kinetics of the two tagged blocks: Distributions of dwell times $p_{1}\left(t\right)$ (not normalized) for the tagged block-chain contacts, with the slopes of the fitted lines indicating the kinetics of contact durations: −1.5 for Block (−2) and −1.0 for Block (−4). Insets display the distributions over longer timescales on a semi-logarithmic scale. (**c**) The time evolution of the inter-contact points for the tagged blocks is shown for Block (−2) on the left and Block (−4) on the right.

In order to gain further insights into the kinetics of the dimer–unimer transition for PAs with varying blockiness, we investigated the dwell times for blocks of different lengths. We labeled a specific minority block in one chain and measured the dwell times of inter-chain contacts with the other chain. Figure 11 demonstrates a few examples of such block dynamics. In dimer states, each block is either bound to the foreign chain (closed state) or unbound (open state), while the overall dimer state is preserved through contacts maintained by other monomers. In the unimer state, all blocks must be in open states simultaneously. Within the dimer state, the probabilities of a specific block with charges of −2,−3, and −4 making contact with the foreign chain are, on average, 0.675, 0.84, and 0.898, respectively. The contact probability for a single isolated charge is approximately 0.45.

We obtained the time distributions $p_{1}\left(t\right)$ by counting the number of switchings from the closed state to the open state within the time interval [t,t+dt], while maintaining the dimer state. This process yields the dwell time distributions for the tagged block and the survival probability of block contacts as defined in Supplementary: $S_{1}\left(t\right)=1-\int_{0}^{t}p_{1}\left(t^{\prime }\right)dt^{\prime }$. It turns out that the dwell time distributions $p_{1}\left(t\right)$ of the closed states follow power laws at short times <100τ, with $p_{1}\left(t\right)∼t^{-\nu }$, with the apparent exponent ν ranging from 1.0 to 1.5 as the labeled block charge changes from (−4) to (−2). (See, Figure 11b). The average dwell times $\langle t_{1}\rangle$ of block contacts for blocks with charges −2,−3, and −4 are approximately 34, 67, and 100τ, respectively, with a time resolution of 5τ. Notably, the increase in dwell time is linear with block length. The dwell time for a single monomer contact is ∼19τ. When we counted events lasting over 50τ, we obtained ∼100, 200, 300τ. The simple variation in dwell time with block length for minority blocks is linked to how easily they can find a complementary sub-sequence of majority type charge.

We also evaluated the dwell times of the majority charge type blocks. The contact durations for blocks with charges of +2, +3, +4, +5, +6, and +7 are 21, 27, 37, 44, 59, and 82τ, respectively. The dwell time for a single monomer contact is approximately 14τ. Notably, the contact times for specific blocks are shorter compared to those with the same charges in the minority type. When we counted events lasting over 50τ, the block dwell times increased by a factor of 7.

The dwell time distributions ultimately decay (approximately) exponentially (Figure 11b). The longer blocks exhibit a more complex behavior, suggesting a power-law decay at intermediate times, though this occurs over a relatively short time regime. This behavior may correspond to the following: as long contact with the other chain is maintained, the block qualitatively slides along the other chain (with a variable number of contacts) in an essentially 1D process, experiencing a random force (without drift). This is reminiscent of the Sinai process [31], which corresponds to a 1/t relaxation of dwell times [32,33], as observed here. The slow relaxation can be qualitatively understood as waiting in front of a barrier. It may be worthwhile to study sequences with even longer blocks to gain further insight.

Simulations confirm that long blocks primarily exhibit sliding before desorbing or switching to their own chain, as illustrated in Figure 11c. A longer charge block (size four) tends to maintain contact with the other chain while the shorter blocks intermittently attach and detach. The longer block explores available sites, shifting to nearby ones. As shown in Figure 11c, the inter-contact points for the long block remain connected over time, continuously shifting. In contrast, the short blocks (size two) make intermittent contact, reattaching at more distant points and displaying hopping dynamics, where the contact points shift in a discontinuous manner.

The dwell times in dimer states (A1) are typically ∼40,000–60,000τ which is over 100 times longer than the single (longest) block contact time. To link it with the dimer duration time, several blocks need to disengage simultaneously from the interaction. We will consider this process under the hypothesis of independent block events. As shown in the Supplementary, for blocks of various lengths, most quantities of interest can be derived from the block dwell time distributions $p_{1}\left(t\right)$ and $p_{2}\left(t\right)$ in closed and open states, characterized by its two first moments. Here, we give a short summary of the results of this toy model. The conditional probability to find the block in a bound/unbound state, starting from a bound/unbound state, relaxes towards the equilibrium population in the target state. The simultaneous dissociation of several independent blocks for the first time has contributions from blocks, pairs of blocks and so on. The single block contributions give a linear combination of relaxation times towards equilibrium bound/unbound distribution corrected by multi-block contributions which can be significant. Even if all considered blocks are unbound the dimer may still hold together due to dispersed monomer-type interactions. Complete dissociation requires that all blocks stay unbound for a time $t_{s}$. This is ruled by survival statistics of the unbound state. It is observed that requiring persistence significantly increases the characteristic time provided that the waiting time divided by the number of blocks exceeds the single block dwell time.

It is tempting to apply a similar argument by considering the block unbinding as the unbinding of its individual monomers. For short blocks, we would expect the unbinding dwell time of the bound state to increase linearly, with corrections for larger blocks.

## 9. Conclusions

By utilizing molecular dynamics simulations and Fokker–Planck type approaches, we investigate the kinetics and theoretical modeling of PA dimerization involving two chains with identical charge distributions and non-vanishing net charges. Our primary focus is examining how the charge sequence influences the dimerization process and the transition kinetics between dimer and unimer states, at a fixed net charge.

The sequence and distribution of charged monomers(residues) along the PA backbone significantly affect the kinetics of dimerization. Particularly, the blockiness of minority charge groups promotes fast and stable dimerization, although the dwell times come with large fluctuations. Variations in charge distribution can lead to substantial differences in the aggregation kinetics, influencing the rate and stability of dimer formation. MD simulations show how PA chains with specific charge sequences with non-vanishing net charges form stable dimers under certain conditions. The stability and formation rate of these dimers are closely correlated with the central net charge (CNC) and the blockiness of the charge sequence. Another important factor is the substantial influence of long majority blocks within the PA chains. These long majority blocks facilitate the formation of extended dimer morphologies, such as dumbbell-like pearl-necklace structures, by enabling the chains to form bridges. This structural characteristic significantly contributes to the duration of the dimer state. In the future, we also plan to study the temporal evolution of aggregate structures beyond dimers to uncover the fundamental principles governing sequence-dependent clustering kinetics.

We employed the Fokker–Planck equation to model the stochastic behavior of the distance between the centers of mass of two unimers. This approach provides an estimate of the average first passage time from the dimer state to the unimer state, as well as in the reverse direction. The probability current and density are derived, imposing spherical symmetry and boundary conditions. The average first passage time is calculated using the Laplace-transformed Fokker–Planck equation, and shows good agreement with simulation results for quasi-spherical unimer shapes.

The dwell time distribution for blocks within a dimer is analyzed, describing transitions between closed (bound) and open (unbound) states. The probability of a block being in an open or closed state over time is modeled, and the long-term behavior of these probabilities is explored. The dwell times in dimer states are significantly longer when multiple blocks are involved, suggesting a cooperative effect in maintaining the dimer state.

Understanding the kinetics of PA dimerization can guide the design of new materials with specific properties. Insights from our study can lead to the development of biocompatible materials with the desired mechanical properties. For example, controlling the rate of coacervate droplet formation could optimize encapsulation efficiency and the release profile of active ingredients in drug delivery systems [9,34]. Additionally, understanding PA aggregation can contribute to the development of hydrogels [35,36,37] and other advanced materials with improved performance.

Our study also provides a better understanding of IDP behavior, which plays a crucial role in cellular functions. The role of physiological ionic strength is not addressed in this work. However, the sequence-specific effects on PA dimerization can inform strategies for modulating polyampholitic protein interactions in biological systems [38].

Our simulations are conducted without explicit solvent, and therefore, hydrodynamic effects are neglected. While the neglect of hydrodynamic effects is not expected to significantly change the overall picture of dimerization/dissociation, we mainly miss two effects: hydrodynamics reduce the friction experienced by a single suspended aggregate, and slow down the ultimate approach into contact with colloidal objects, particularly at distances much smaller than the relevant colloidal radius. To account for hydrodynamics, the friction in the free-draining limit should be replaced with the reduced friction applied in the non-draining limit. In the non-draining limit, the friction typically exhibits moderate shape dependence. A more pronounced effect of shape anisotropy is expected for pearl-necklace-type structures.

The data accumulated on PA aggregation and dimerization kinetics illustrate processes that await experimental investigations. It is likely that some experimental techniques used for micelles [39] could be adapted to the specific case of PA aggregates. These include spectrometric techniques [40], such as Förster Resonance Energy Transfer (FRET) [41] or scattering techniques [42,43].

In conclusion, the combined analysis offers a comprehensive understanding of the kinetics and theoretical aspects of PA dimerization. The findings highlight the critical role of charge sequence including blockiness in determining the aggregation behavior of PAs.

## Figures and Tables

**Table 1:** Grouping according to the blockiness of minority of charges for ensemble of *Q* = 8.

Group	Minority Block Size	Percentage ^a^	Sequences
AAA	(−6)×1	4%	44, 47
AA	(−5)×1	2%	26
A2	(−4)×2	2%	46
A1	(−4)×1	24%	1, 13, 18, 19, 24, 28, 35, 39, 40, 42, 45, 50
	(−4)×1,(−3)×1	(10%)	24, 43, 18, 35, 40
	(−4)×1,(−2)×n	(14%)	1, 13, 19, 28, 39, 42, 45, 50
B3	(−3)×3	6%	16, 17, 29
B2	(−3)×2	14%	2, 4, 5, 23, 27, 36, 41
B1	(−3)×1	24%	3, 7, 8, 10, 11, 14, 21, 33, 34, 37, 48, 49
C5	(−2)×5	2%	31
C4	(−2)×4	8%	6, 9, 22, 43
C3	(−2)×3	14%	12, 15, 20, 25, 30, 32, 38

^a^ The percentages being even numbers, such as 2% or 4%, do not carry significant meaning; this is simply a result of the sample size of 50.

## Data Availability

The original contributions presented in the study are included in the article/Supplementary Material, further inquiries can be directed to the corresponding authors.

## Supplementary Materials

The following supporting information can be downloaded at: https://www.mdpi.com/article/10.3390/polym16202928/s1, including the theory, molecular dynamics description, and data: Figure S1: Representative snapshots of unimer association and dimer dissociation processes; Figure S2: Potential profiles for dimerization in center-to-center coordinates; Table S1: Eigenvalues of moment of inertia tensor; Videos S1–S4: Unimer association and dimer dissociation processes. References [26,44] are cited in the supplementary materials.
