# Supplementary material for: Kinetics of Polyampholyte Dimerization: Influence of Charge Sequences
Source: Polymers (Basel). 2024 Oct 18;16(20):2928. doi: 10.3390/polym16202928 (PMC11510756; doi:10.3390/polym16202928)
Supplement: Supplementary file 1 [file polymers-16-02928-s001.zip › polymers-3245631-supplemantary.pdf]

Supplementary Materials: Kinetics of Polyampholyte Dimerization: Influence of Charge Sequences

Seowon Kim [1], Nam-Kyung Lee [1,*], Youngkyun Jung [2], and Albert Johner [3,*]

1. Theory

1.1. Dimerization Kinetics from the Statistics of the Distance between the Centers of Mass of the Two Unimers: Fokker-Planck Type Approach

The random variable considered is the center to center distance **r** of the two unimers. The conservation law of the probability density P(**r**) reads:

$$\frac{\partial P}{\partial t} + \nabla \cdot \mathbf{j} = 0 \tag{S1}$$

where the probability current **j** is here:

$$\mathbf{j} = -D\frac{\partial P}{\partial \mathbf{r}} - DP\frac{\partial V}{\partial \mathbf{r}} \tag{S2}$$

The friction enters the problem when the centers of mass of both unimers move and experience position fluctuations. The center-to-center distance experiences the double fluctuation and hence the diffusion constant D above is taken twice that of one unimer. We assume that the probability has spherical symmetry and only depends on the radial coordinate r rather than on **r**.

The average time for going from the dimer state to the unimer state can be estimated from the Fokker-Planck (FP) equation. We consider the system at the free energy minimum (shell) on the dimer side at location r_2 (state (2)) and calculate its average first passage time at the minimum on the unimer side (state (1)) located at position r_1 (Fig. 7). We choose to proceed with the Laplace transformed Fokker-Planck (FP) equation with s the conjugate of Dt.

$$s\tilde{P} - P(t=0) + \nabla_r \cdot \tilde{j} = 0 \tag{S3}$$

Here and below $\tilde{f}(s)$ designates the Laplace transform (LT) of $f(t)$. For a start in state (2) : $P(t=0) = \frac{\delta(r-r_2)}{4\pi {r_2}^2}$, further state (1) is adsorbing, $P(r_1) = 0$, to describe the first passage time statistics at state (1).

To assess the moments of the first passage time distribution we expand $\tilde{P}$ and $\tilde{j}$ in powers of s around $s = 0$. $\tilde{P} = P_0 + sP_1 + ...$ and $\tilde{j} = j_0 + sj_1 + ...$ The average first passage time at state (1) is defined as $\langle t \rangle = \int_0^\infty t 4\pi r_1^2 j(r_1)$ and obtained as $-4\pi r_1^2 j_1(r_1)$.

The first terms of the expansions of $\tilde{j}$ and $\tilde{P}$ follow from the LT of the FP equation. The lowest order current j_0 conserves flux on either side of the shell of radius r_2. No probability flows into $r = 0$, all probability ultimately flows into the adsorbing shell of radius r_1. As a consequence, $j_{0<} = 0, (r < r_2)$, $j_{0>} = \frac{1}{4\pi r^2}, (r > r_2)$, which also ensures the proper discontinuity on the r_2-shell. From this, P_0 is obtained by integration of the first order equation defining the probability current where $\nabla \leftrightarrow \frac{\mathrm{d}}{\mathrm{d}r} + \frac{2}{r}$. As a result, we have: $P_{0<}(r) = -e^{-V(r)} \int_{r_1}^{r_2} \frac{e^{V(r')}}{4\pi r'^2} \mathrm{d}r'$ and $P_{0>} = -e^{-V(r)} \int_{r_1}^{r} \frac{e^{V(r')}}{4\pi r'^2} \mathrm{d}r'$, where we have taken into account that $P_{0>}(r_1) = 0$ and the continuity of P_0 at r_2. Note the Boltzmann type distribution in the no current region ($r < r_2$) and that the probability density vanishes beyond the adsorbing shell enclosing the source.

The next order term, j_1, in the current is obtained from the FP equation in the same way as j_0, but with the Dirac distribution source term replaced by sP_0. The expression for $j_1(r)$ is given by $j_1(r) = \frac{1}{r^2} \int_0^r P_0(r') r'^2 \mathrm{d}r'$, where the lower bound in the integral is set to

zero to avoid singularities at 0. The sought mean first passage time (dimer dwell time) is obtained as:

$$\langle t_d \rangle = \int_0^{r_1} P_0(r') 4\pi r'^2 \mathrm{d}r', \tag{S4}$$

where P_0 is piecewise defined above.

In the same way we may calculate the first passage time at state (2) when starting in state (1). In contrast to the previous case the adsorbing shell of radius r_2 does not enclose the source on the shell of radius r_1 (also the center $r = 0$ is screened by the the adsorbing shell). We hence introduce a reflecting shell at $\mathcal{L}$ enclosing the system. Making the proper adjustments we arrive at $P_{0>}, (r > r_1)$, $P_{0<}, (r < r_1)$, along with the expression for the mean first passage time (unimer dwell time):

$$P_{0>}(r) = e^{-V(r)} \int_{r_2}^{r_1} \frac{e^{V(r')}}{4\pi r'^2} \mathrm{d}r'; \ (r > r_1) \tag{S5}$$

$$P_{0<}(r) = e^{-V(r)} \int_{r_2}^{r} \frac{e^{V(r')}}{4\pi r'^2} \mathrm{d}r'; \ (r < r_1) \tag{S6}$$

$$\langle t_u \rangle = \int_{r_2}^{\mathcal{L}} P_0(r') 4\pi r'^2 \mathrm{d}r'. \tag{S7}$$

1.2. *Dimer dwell time and bound/unbound block transitions: a toy model*

We consider a block initially bound to the other chain inside a dimer and want to describe its state either closed (bound to the other chain) or open (free or bound to its own chain) at time t. This is done in terms of the dwell time distribution in either state $p_1(t)$ for the closed state and $p_2(t)$ in the open state. Using LT with respect to time (see above) to reduce convolutions to ordinary products, the probability for the block to be in the open state and close state when starting out from the close state read respectively:

$$\tilde{p}_{(c|c)} = \frac{1}{s}(1 - \tilde{p}_1)\frac{1}{1 - \tilde{p}_1\tilde{p}_2} \quad \text{and} \quad \tilde{p}_{(o|c)} = \frac{1}{s}(1 - \tilde{p}_2)\frac{\tilde{p}_1}{1 - \tilde{p}_1\tilde{p}_2}. \tag{S8}$$

The final state is either closed or open and these two probabilities add up to $1/s$ (1 in direct space). From the simulations we obtained the two first moments of the dwell time distributions which yield the large time/small expansions: $\tilde{p}_1 = 1 - s\langle t_1 \rangle + \frac{s^2}{2}\langle t_1^2 \rangle$ and similarly for $\tilde{p}_2$ with index 2 in place of index 1. We are interested in the long time/small-s limits which can be cast in the form:

$$\tilde{p}_{(o|c)} = \frac{\langle t_2 \rangle}{s(\langle t_1 \rangle + \langle t_2 \rangle)} - \frac{2\langle t_1 \rangle^2 \langle t_2 \rangle + \langle t_1 \rangle \langle t_2^2 \rangle - \langle t_1^2 \rangle \langle t_2 \rangle}{2(\langle t_1 \rangle + \langle t_2 \rangle)^2} + O(s). \tag{S9}$$

The next order $\sim s$ involves the $\sim s^3$ term in the $\tilde{p}_1$, $\tilde{p}_2$ expansion. Provided the constant term in Eq. S9 is negative, which is likely, the expansion can be cast as $\tilde{p}_{(o|c)} \sim \frac{\langle t_2 \rangle}{\langle t_1 \rangle + \langle t_2 \rangle} \frac{\tau_c^{-1}}{s(\tau_c^{-1} + s)}$ or in direct space:

$$p_{(o|c)}(t) \sim \frac{\langle t_2 \rangle}{\langle t_1 \rangle + \langle t_2 \rangle}\left(1 - e^{-t/\tau_c}\right)$$

$$\tau_c = \frac{2\langle t_1 \rangle^2 \langle t_2 \rangle + \langle t_1 \rangle \langle t_2^2 \rangle - \langle t_1^2 \rangle \langle t_2 \rangle}{2(\langle t_1 \rangle + \langle t_2 \rangle)\langle t_2 \rangle}. \tag{S10}$$

The asymptotic value corresponds to the equilibrium probability P_{eq2} of the open state according to ergodicity while the final relaxation time τ_c is a combination of the first two moments of the dwell time distributions p_1 and p_2. If the constant term in Eq. S9 were positive, the above would apply to $\tilde{p}_{(c|c)} = 1/s - \tilde{p}_{(o|c)}$. Only the approach to the asymptote is described here. As $p_{(c|c)}$ starts out from unity and approaches its asymptote from below, it would exhibit an undershoot before settling at the asymptotic value. Similar forms are

obtained for other conditional probabilities and the equilibrium probability only depends on the final state whilst the relaxation functions need to be explicitly calculated. Most important here are $p_{(o|c)}$ and $p_{(o|o)}$.

$$\tilde{p}_{(o|o)} = \frac{\langle t_2\rangle}{s(\langle t_1\rangle + \langle t_2\rangle)} + \frac{2\langle t_1\rangle\langle t_2\rangle^2 - \langle t_1\rangle\langle t_2^2\rangle + \langle t_1^2\rangle\langle t_2\rangle}{2(\langle t_1\rangle + \langle t_2\rangle)^2} + O(s). \tag{S11}$$

Typically the constant is expected to be positive. As a consequence, we obtain $p_{(o|o)}$ from $p_{(c|o)}$ as:

$$p_{(o|o)} = \frac{\langle t_2\rangle}{\langle t_1\rangle + \langle t_2\rangle} + \frac{\langle t_1\rangle}{\langle t_1\rangle + \langle t_2\rangle} \exp -t/\tau_o \tag{S12}$$

where:

$$\tau_o = \frac{2\langle t_1\rangle\langle t_2\rangle^2 + \langle t_2\rangle\langle t_1^2\rangle - \langle t_2^2\rangle\langle t_1\rangle}{2(\langle t_1\rangle + \langle t_2\rangle)\langle t_1\rangle}. \tag{S13}$$

It so happens that asymptotic expressions for both $p_{(o|c)}$ and $p_{(o|o)}$ also give the correct value for $t = 0$ and may give a fair description over the whole t-range. However, the expressions for τ_c (Eq. S10) and τ_o (Eq. S12) are not immediately transparent. To fix the idea, we may refer to exponential distributions, where the average of the square is twice the square of the average. This relation approximately holds for the simulation data (though we are not yet implying that exponential distributions for the block dwell times) and leads to the much simpler expression: $1/\tau_c = 1/\tau_o = 1/\langle t_1\rangle + 1/\langle t_2\rangle$.

Consider a dimer with all its n blocks initially closed. Assuming that the blocks are statistically independent, the probability for them to be all open at time t reads: $P_{\{(o|c)\}}(t) = \Pi_{i=1}^{n} P_{eq2|i}(1 - e^{-t/\tau_i})$. We will come back on this assumption below. The first passage time distribution for all blocks being open $\tilde{P}^f_{\{(o|c)\}}$ obtains from the Dyson equation. In LT:

$$\tilde{P}^f_{\{(o|c)\}} = \frac{\tilde{P}_{\{(o|c)\}}}{1 + \tilde{P}_{\{(o|o)\}}} \tag{S14}$$

where $P_{\{(o|o)\}}(t)$ is the conditional probability that all blocks are again open after time t. At asymptotically large times the first passage event contributes little to the overall passage and we expect 1 to be negligible in the denominator of Equation (S14). Conversely, if we were to keep only the term 1, the associated average first passage time would diverge. As the direct space terms are all exponential in the numerator and denominator in Equation (S14), it becomes straightforward to proceed and get $\tilde{P}^f_{\{(o|c)\}}$. Below we give the average first passage time $\langle t_f\rangle$ when all blocks are open simultaneously:

$$\begin{aligned}\langle t_f\rangle = &\sum_{\text{blocks}} \tau_c^{(i)} - \sum_{\text{pairs of blocks}} (1/\tau_c^{(i)} + 1/\tau_c^{(j)})^{-1} + \ldots + \\ &\sum_{\text{blocks}} \frac{\langle t_1\rangle^{(i)}}{\langle t_2\rangle^{(i)}}\tau_o^{(i)} + \sum_{\text{pairs of blocks}} \frac{\langle t_1\rangle^{(i)}}{\langle t_2\rangle^{(i)}}\frac{\langle t_1\rangle^{(j)}}{\langle t_2\rangle^{(j)}}(1/\tau_o^{(i)} + 1/\tau_o^{(j)})^{-1} + \ldots\end{aligned} \tag{S15}$$

The sum in the first line is alternating and generated by the numerator in Eq. S14, while the sum in the last line is not alternating, and is generated by the denominator, where the first term (1) is neglected. The time $\langle t_f\rangle$ is not necessarily dominated by the first term of either sum.

It is more natural to start out from the equilibrium distribution of closed and open states which leads to the average first passage time at the all-open state:

$$\begin{aligned} \langle t_f \rangle = & \sum_{\text{blocks}} \frac{\langle t_1 \rangle^{(i)}}{\langle t_1 \rangle^{(i)} + \langle t_2 \rangle^{(i)}} (\tau_c^{(i)} - \tau_o^{(i)}) \\ & - \sum_{\text{pairs of blocks}} \frac{\langle t_1 \rangle^{(i)}}{\langle t_1 \rangle^{(i)} + \langle t_2 \rangle^{(i)}} \frac{\langle t_1 \rangle^{(j)}}{\langle t_1 \rangle^{(j)} + \langle t_2 \rangle^{(j)}} \times \tau_{ij} + \ldots \\ & + \sum_{\text{blocks}} \frac{\langle t_1 \rangle^{(i)}}{\langle t_2 \rangle^{(i)}} \tau_o^{(i)} + \sum_{\text{pairs of blocks}} \frac{\langle t_1 \rangle^{(i)}}{\langle t_2 \rangle^{(i)}} \frac{\langle t_1 \rangle^{(j)}}{\langle t_2 \rangle^{(j)}} (1/\tau_o^{(i)} + 1/\tau_o^{(j)})^{-1} + \ldots \end{aligned} \tag{S16}$$

where $\tau_{ij} = (1/\tau_c^{(i)} + 1/\tau_c^{(j)})^{-1} + (1/\tau_o^{(i)} + 1/\tau_o^{(j)})^{-1} - (1/\tau_o^{(i)} + 1/\tau_c^{(j)})^{-1} - (1/\tau_c^{(i)} + 1/\tau_o^{(j)})^{-1}$ and the last line generated by the denominator in Eq. S14 is unchanged. Note that among the terms listed in Eq. S16, only the contribution of the denominator survives for exponential block dwell time distributions.

We use operational definitions of the dimer state and unimer state which are based on the number of contacts (simulations) or on the separation between the centers of mass of the two unimers (FP equation). When all blocks are open for the first time it is very likely that one of them closes within a very short time span. If we do not want to count these very short lived dissociated states which may further be held by simple monomer contacts, we may ask for the all-open state to last at least for a time t_S. For each block this criterion involves the survival probability S_o in the open state, $S_o(t) = 1 - \int_0^t p_2(t')\mathrm{d}t'$. The long time asymptotics of $S_o(t)$ reads $S_o(t) \sim \frac{2\langle t_2 \rangle^2}{\langle t_2^2 \rangle} \exp\left(-\frac{2\langle t_2 \rangle}{\langle t_2^2 \rangle} t\right)$. This asymptotic law does not extrapolate to unity for vanishing time except for exponential $p_2(t)$ distribution, where the characteristic decay time further reduces to $\langle t_2 \rangle$. Assuming that the blocks are independent, the probability for all blocks to remain open after time t_S is the product of the individual survival probabilities.

$$\prod_i S_o^i = \left(\prod_i \frac{2(\langle t_2 \rangle^{(i)})^2}{\langle (t_2)^2 \rangle^{(i)}} \right) \exp(-t_S \sum_i \frac{2\langle t_2 \rangle^{(i)}}{\langle t_2^2 \rangle^{(i)}}) \tag{S17}$$

where the index (i) runs over the blocks. Depending on the prescribed time t_S a large multiplicative factor may apply to $\langle t_f \rangle$ given in Equations (S15) and (S16).

Throughout we assumed blocks to behave independently. In the very opposite case of strongly coupled blocks, longer blocks may trigger the switching of shorter ones and the global opening time would be reduced. Long blocks dominate the opening dynamics in both independent and coupled blocks. Results should remain similar.

2. Molecular Dynamics Simulation Description

In the molecular dynamics simulations, we consider a system of PA chains with a well defined charge sequence in weakly poor solvent condition, with no added salt. Each PA is modeled as a bead-spring chain consisting of N = 100 monomers, each with a diameter σ (approximately equal to the average bond length b). All considered sequences for the dimerization study bear net charges of Q = 8. The electro-neutrality is imposed by counterions compensating the net charge on the PA backbones. Counterion condensation effect does not play a major role in the density condition we considered, albeit some charge regulation occurs.

The motion of beads is described by the following equation with the total energy $U = U_{\rm LJ} + U_{\rm C} + U_{\rm FENE}$,

$$m\frac{d\mathbf{v}_i(t)}{dt} = -\zeta\frac{\partial \mathbf{r}_i}{\partial t} - \frac{dU}{d\mathbf{r}_i} + \mathbf{f}^R(t),$$

where ζ is the frictional coefficient and m is the mass of the bead. Here, $\mathbf{r}_i$ and $\mathbf{v}_i$ are the position and the velocity of particle i, respectively. The Gaussian random force $\mathbf{f}^R$ has zero average $\langle \mathbf{f}^R(t)\rangle = 0$ and correlations $\langle \mathbf{f}^R(t)\cdot\mathbf{f}^R(t')\rangle = 6k_{\rm B}T\zeta\delta(t-t')$ set the temperature of the system. The temperature is set to $T = T_0$ such that $k_{\rm B}T_0 = 1$ and we measure energy in thermal unit $k_{\rm B}T_0$. Additionally, we set ζ = 1.0 $m\tau^{-1}$ where $\tau = (m\sigma^2/k_{\rm B}T)^{1/2}$ is the characteristic time scale. Two charged particles interact via the Coulomb potential $U_{\rm C}(r_{ij}) = z_i z_j \frac{l_{\rm B}}{r_{ij}} k_{\rm B}T$, where z_i and z_j are the charge valence of particle i and j, and r_{ij} denotes the center-to-center distance between i and j particles. The strength of electrostatic interactions is determined by the Bjerrum length $l_{\rm B}$. In our simulations for PAs, we set $l_{\rm B} = 3\sigma$ at T_0. The long-range electrostatic interactions are calculated by the particle-particle-particle-mesh (PPPM) method implemented in LAMMPS software package [1].

The chain connectivity is ensured by the finite extension nonlinear elastic (FENE) potential $U_{\rm FENE}$ between two consecutive beads [2] $U_{FENE}(r) = -0.5kr_0^2\ln[1-(r/r_0)^2]$, where the spring constant is taken as $k = 30k_{\rm B}T/\sigma^2$ and the maximum bond length as $r_0 = 1.5\sigma$. With this potential, the equilibrium bond length is 1.004±0.002 σ, indicating minimal deviation from the average bond length.

The excluded volume interactions are modeled by the truncated-shifted Lennard-Jones(LJ) potential: $U_{\rm LJ}(r_{ij}) = 4\epsilon_{\rm LJ}[(\sigma/r_{ij})^{12} - (\sigma/r_{ij})^6 - (\sigma/r_c)^{12} + (\sigma/r_c)^6]$ for $r_{ij} < r_c$ and 0 elsewhere. Here $\epsilon_{\rm LJ}$ represents the strength of the LJ potential. In simulations of random PAs, the value of the interaction parameter is set to $\epsilon_{\rm LJ} = 0.6$ and the cutoff distance r_c is set to 2.5σ for monomer-monomer interactions so that polymers are in moderately poor solvent condition. For monomer–counterion and counterion–counterion interactions, we set the interaction parameter $\epsilon_{\rm LJ} = 1.0$ and the cut off distance $2^{1/6}\sigma$, which leads to purely repulsive interactions.

We integrated the equation of motion using the velocity Verlet algorithm with an integration time step $\delta t = 0.005\tau$. We first performed 10^7 integration steps (= $5 \times 10^4\tau$) in order for the mean square radius of gyration of the chain to relax to its equilibrium values for two chain simulations. After equilibration, we ran additional 2.4×10^8 integration steps (= $1.2 \times 10^6\tau$) and collected data every 10^3 time steps (= 5 τ) for two chain simulations.

3. Kinetics of PA Dimerization

3.1. Moment of Inertia Tensor, Eigenvalues

To study the shapes of a dimer consisting of $2N$ monomers, we calculate the three principal moments of inertia for a given configuration of the dimer, which are obtained from the moment of inertia tensor

$$I_{ij} = \frac{1}{2N} \sum_{\alpha=1}^{2N} m_\alpha \left[\delta_{ij} \left(\sum_{k=1,2,3} x_{\alpha k}^2 \right) - x_{\alpha i}\, x_{\alpha j} \right],$$

where $i, j = 1, 2, 3$ and $x_{\alpha k}$ represents the position of a α-monomer relative to the center of mass of the dimer.

We recorded three real eigenvalues, denoted as $\lambda_1 \leq \lambda_2 \leq \lambda_3$, at a given time from the moment of inertia tensor. The time averaged values are reported in Fig. 2, where $\lambda_3(\lambda_1)$ represents the time-averaged value of the largest (smallest) eigenvalue.

Table S1. Eigenvalues λ_1,λ_2, and λ_3 for the conformations shown in Fig. 1. In (a) and (b), the conformations are labeled as 1, 2, and 3 from left to right.

Sequence	timestep	λ_1	λ_2	λ_3
17	a-1	6.08	19.18	20.31
	a-2	6.19	22.28	22.80
	a-3	8.83	9.52	13.26
29	b-1	8.20	9.09	12.21
	b-2	6.90	20.58	23.52
	b-3	7.67	12.94	16.79
46	c	7.43	10.65	11.99

3.2. Dimerization and Dissociation

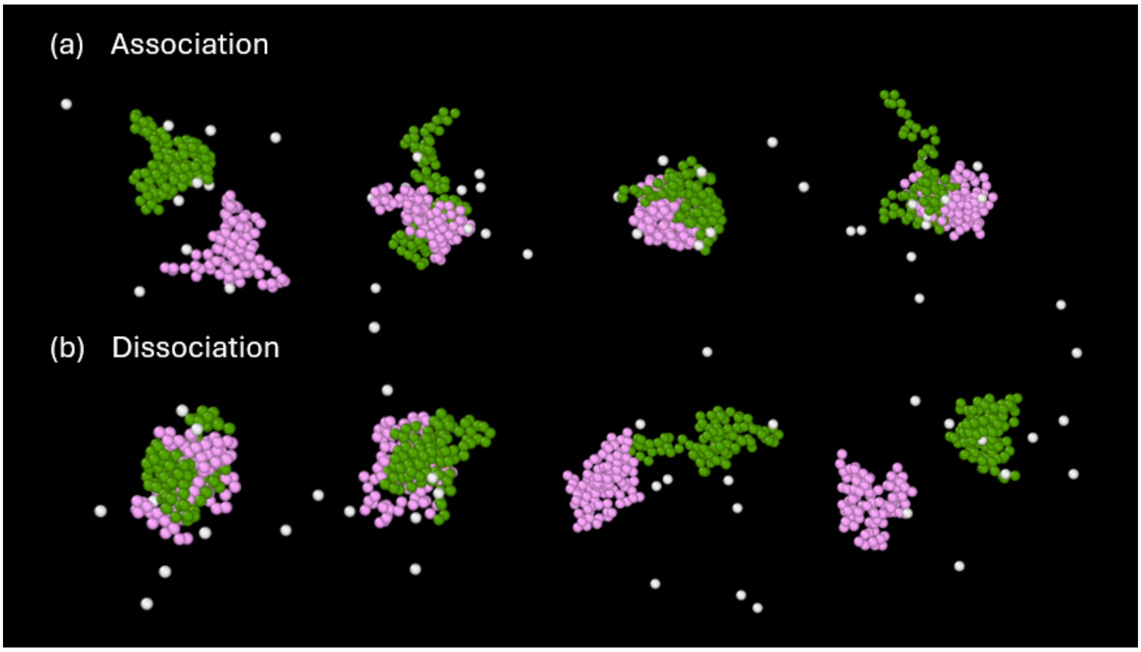

Figure S1. Snapshots illustrating association and dissociation processes of two PA chains (seq.39). Each shown process spans approximately 3000τ. The two unimers are distinguished by pink and green colors. White spheres indicate counterions.

3.3. Potential Profiles

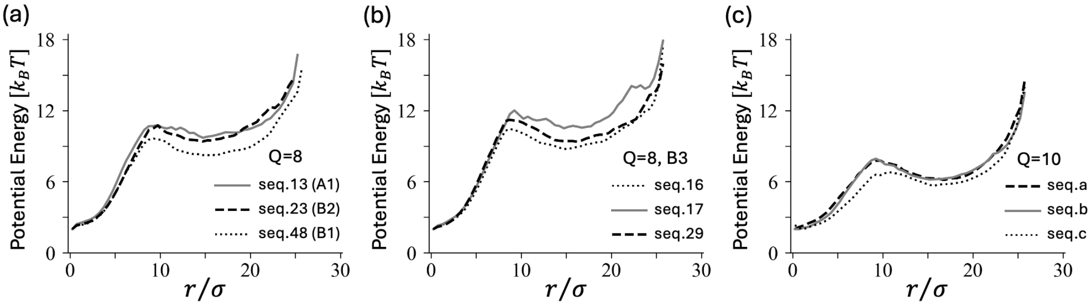

(d)

Q=10

Seq	Minority Blocks	Majority Blocks	1	2	3	4	5	6	7	8	9	10	11	12	13
a	(-4)*1,(-3)*1	(7)*1,(6)*1,(3)*2	1	-1	1	1	1	-1	1	1	1	1	1	1	1
b	(-2)*3	(4)*1,(3)*2	1	1	-1	1	1	-1	-1	1	1	-1	1	1	1
c	(-2)*3	(5)*1,(4)*1,(3)*3	1	-1	1	1	1	-1	1	1	1	1	1	-1	-1

Seq	14	15	16	17	18	19	20	21	22	23	24	25	26	27	28	29	30	31	32	33	34
a	-1	-1	-1	1	-1	1	-1	1	1	1	1	1	1	-1	-1	-1	-1	1	1	1	-1
b	1	-1	1	1	-1	-1	1	-1	1	1	1	-1	1	-1	1	1	-1	-1	1	1	1
c	1	-1	1	1	1	1	-1	1	-1	-1	1	1	1	-1	1	1	1	-1	1	-1	-1

Figure S2. (a) Potential profiles for representative sequences of Q = 8 and CNC = 5, seq.13 (A1, $(-4) \times 1$,$(-2) \times 3$), seq.23 (B2,$(-3) \times 2$,$(-2) \times 1$) and seq.48(B1,$(-3) \times 1$,$(-2) \times 2$). (b) Potential profiles for three sequences, seq.16, seq.17, and seq.29 belonging to Group B3 ($(-3) \times 3$). The CNC values for these sequences are 4, 5, and -1, respectively. (c) The landscape of three sequences with Q = 10 are shown for comparison: sequence a ($(-4) \times 1$) and sequence b and c ($(-2) \times 3$). (d) These three sequences from (c) are shown below. The center-to-center distances r are chosen as reaction coordinates.

We evaluate the potential profiles for three sequences that share the same CNC value (CNC = 5) but exhibit varying levels of blockiness. Sequence 13, characterized by high blockiness, displays the slowest switching kinetics (long dissociation times), which corresponds to the largest energy barrier for dissociation. This analysis of sequences with the same CNC value but different blockiness levels underscores the significant impact of block structure on switching kinetics. Furthermore, we compare the potential profiles of three sequences within the same B3 group but with different CNC values. The unimer and dimer states of seq.29, with CNC = -1, are expected to be more compact and stable, leading to a higher energy barrier. Finally, the energy landscapes of three sequences with larger net charges (Q = 10) are noticeably shifted, indicating a preference for unimer states.

References

1. Plimpton, S. Fast parallel algorithms for short-range molecular dynamics. *J. Comput. Phys.* **1995**, *117*, 1–19. Also see http://lammps.sandia.gov.
2. Chae, M. -K.; Lee, N. -K.; Jung, Y.; Johner, A.; Joanny, J. F. Partially globular conformations from random charge sequences. *ACS Macro Lett.* **2022**, *11*, 382-386.
