# Supplementary figures and images for: Kinetics of Polyampholyte Dimerization: Influence of Charge Sequences
Source: Polymers (Basel). 2024 Oct 18;16(20):2928. doi: 10.3390/polym16202928 (PMC11510756; doi:10.3390/polym16202928)

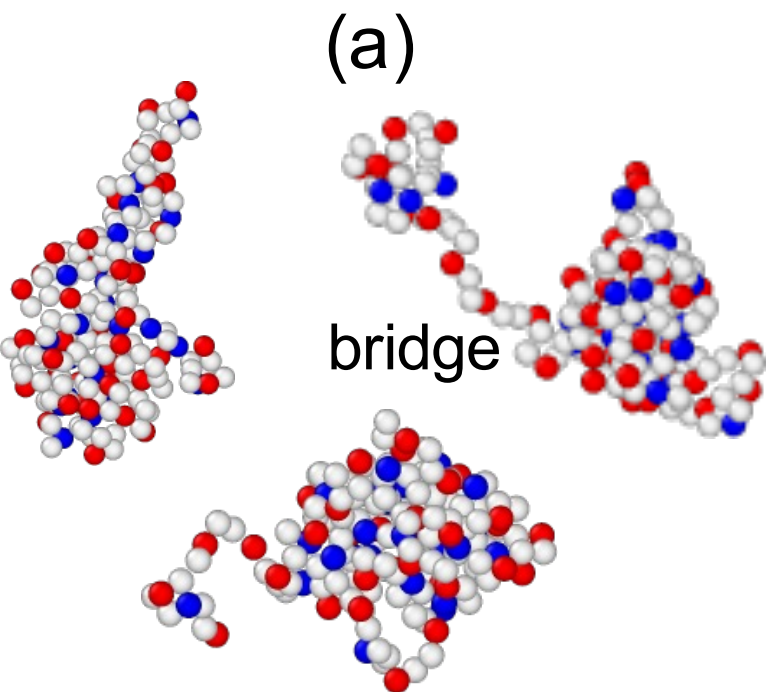

$(-3) \times 3$  (seq.17)

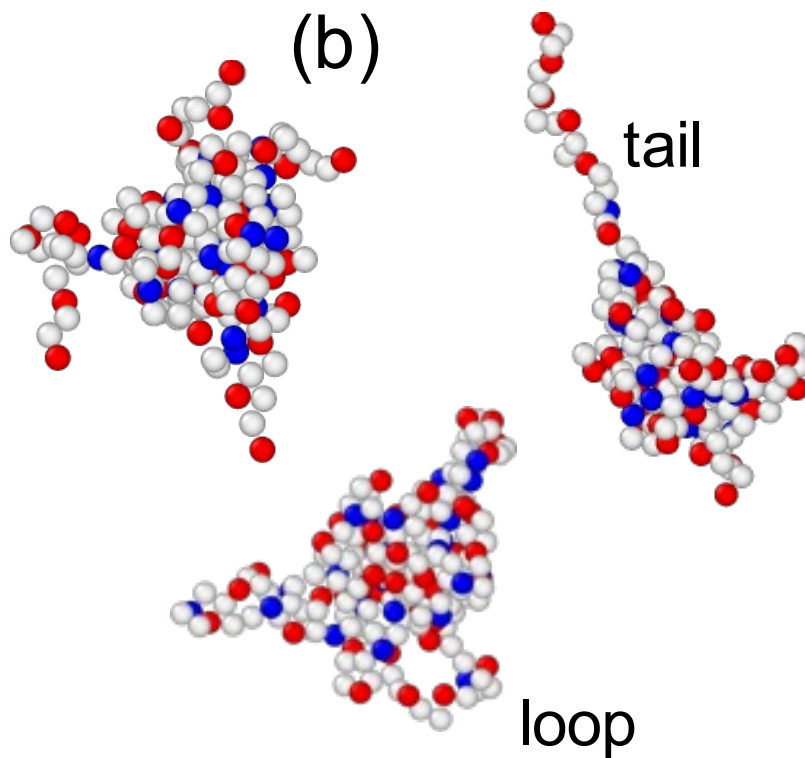

$(-3) \times 3$  (seq.29)

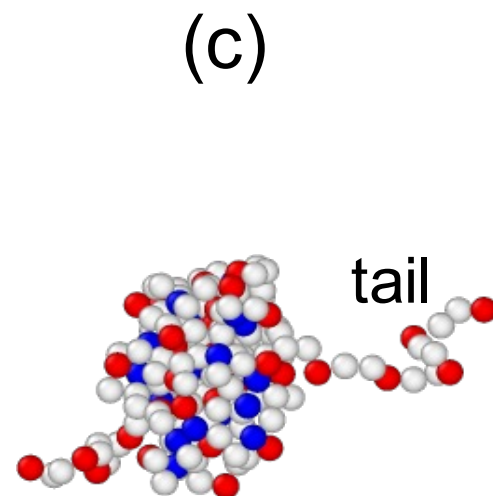

$(-4) \times 2$  (seq.46)

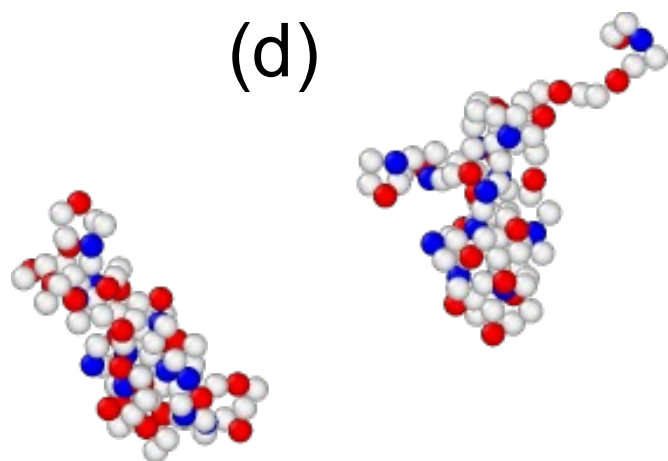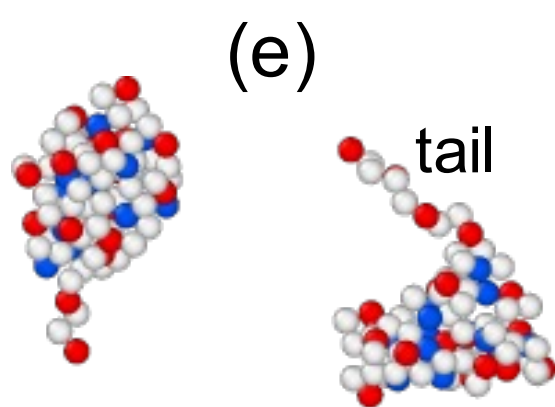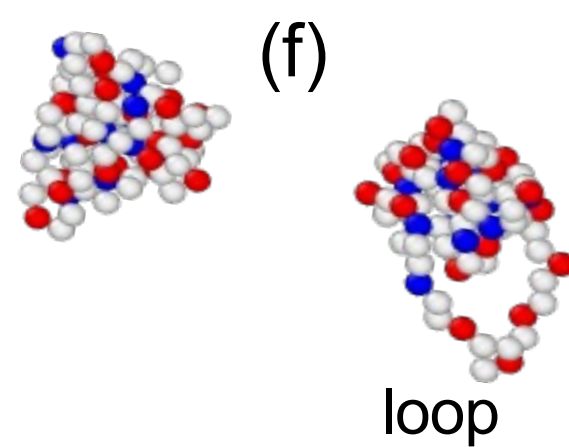

Supplement: Supplementary file 1 [file polymers-16-02928-s001.zip › Definitions/Fig1.pdf]

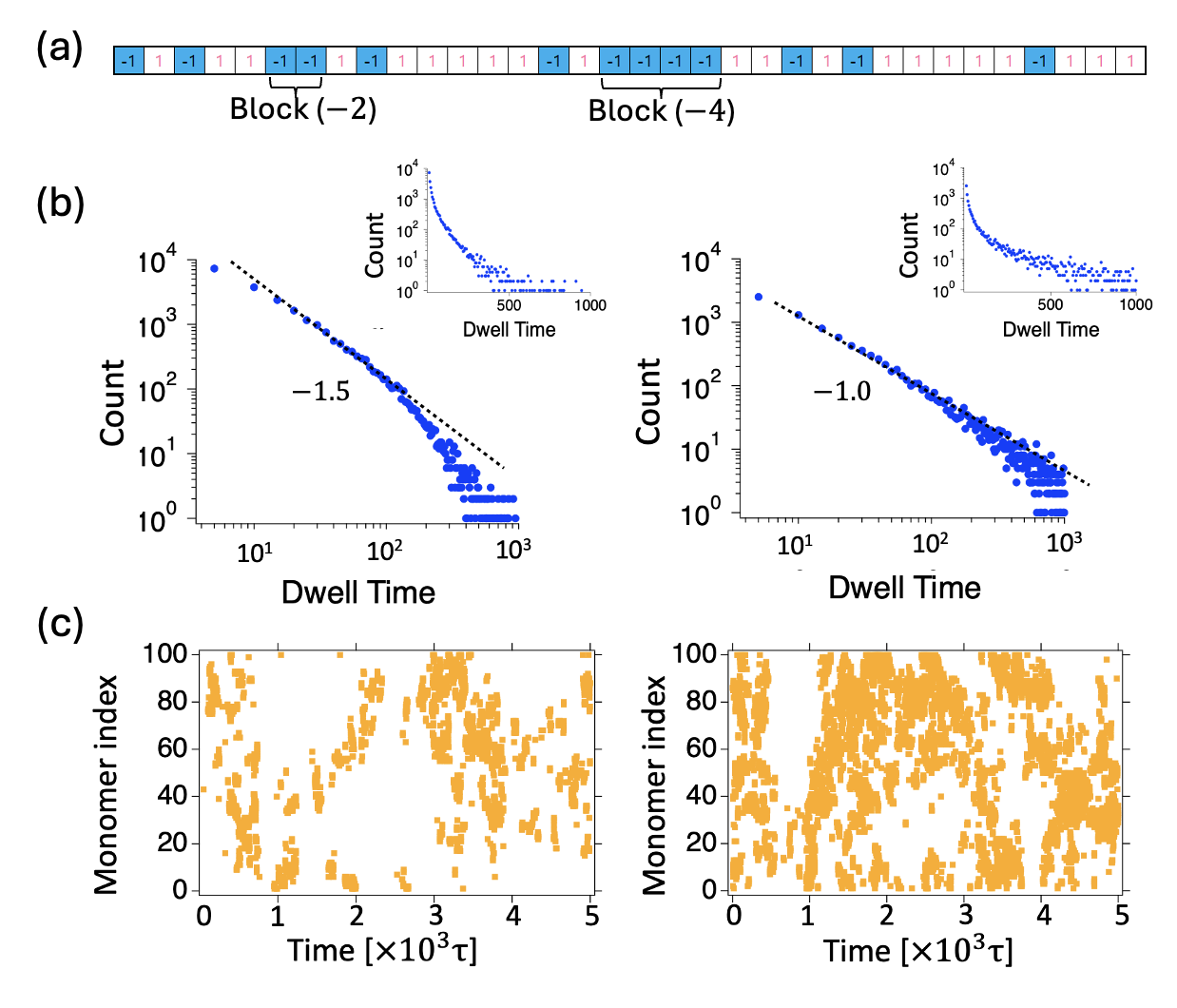

Supplement: Supplementary file 1 [file polymers-16-02928-s001.zip › Definitions/Fig10block.png]

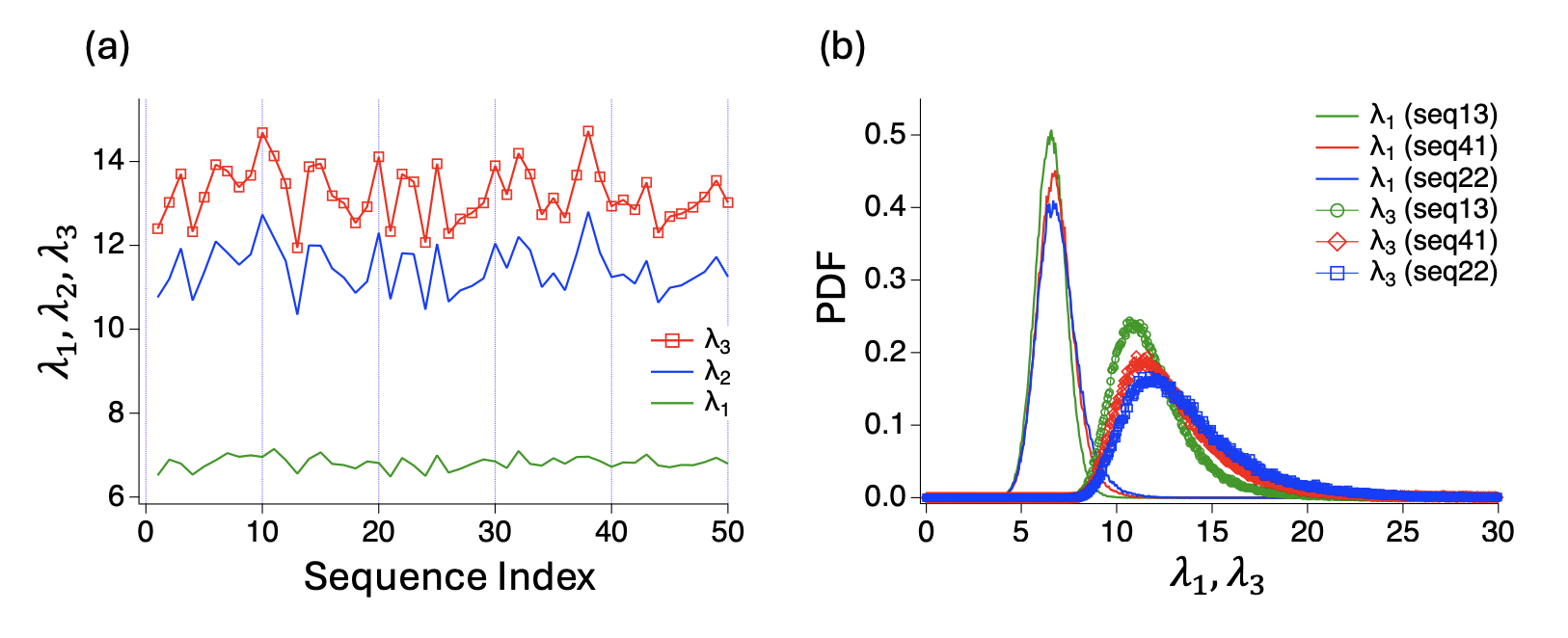

Supplement: Supplementary file 1 [file polymers-16-02928-s001.zip › Definitions/Fig2Inertia.png]

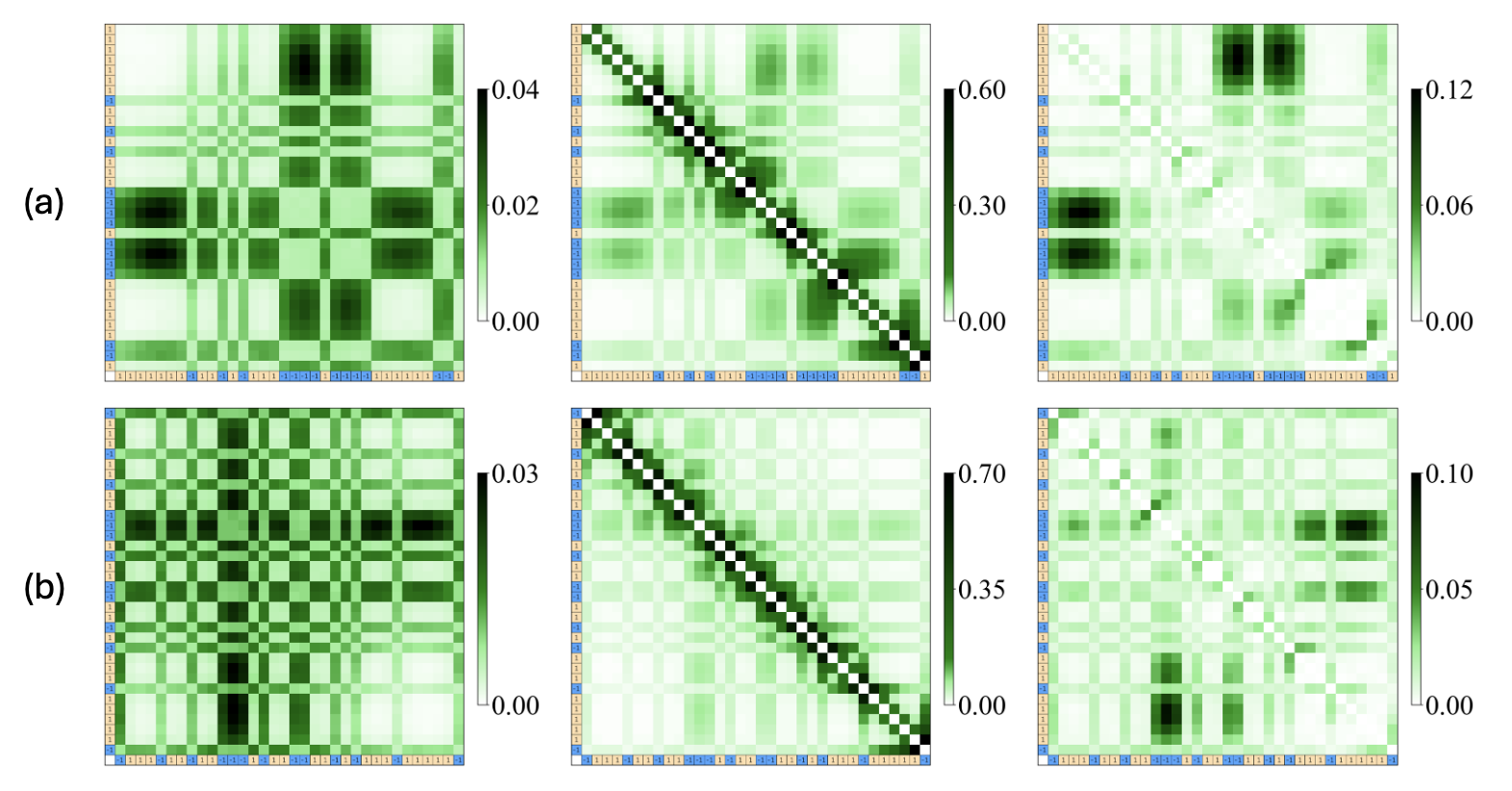

Supplement: Supplementary file 1 [file polymers-16-02928-s001.zip › Definitions/Fig3CM.png]

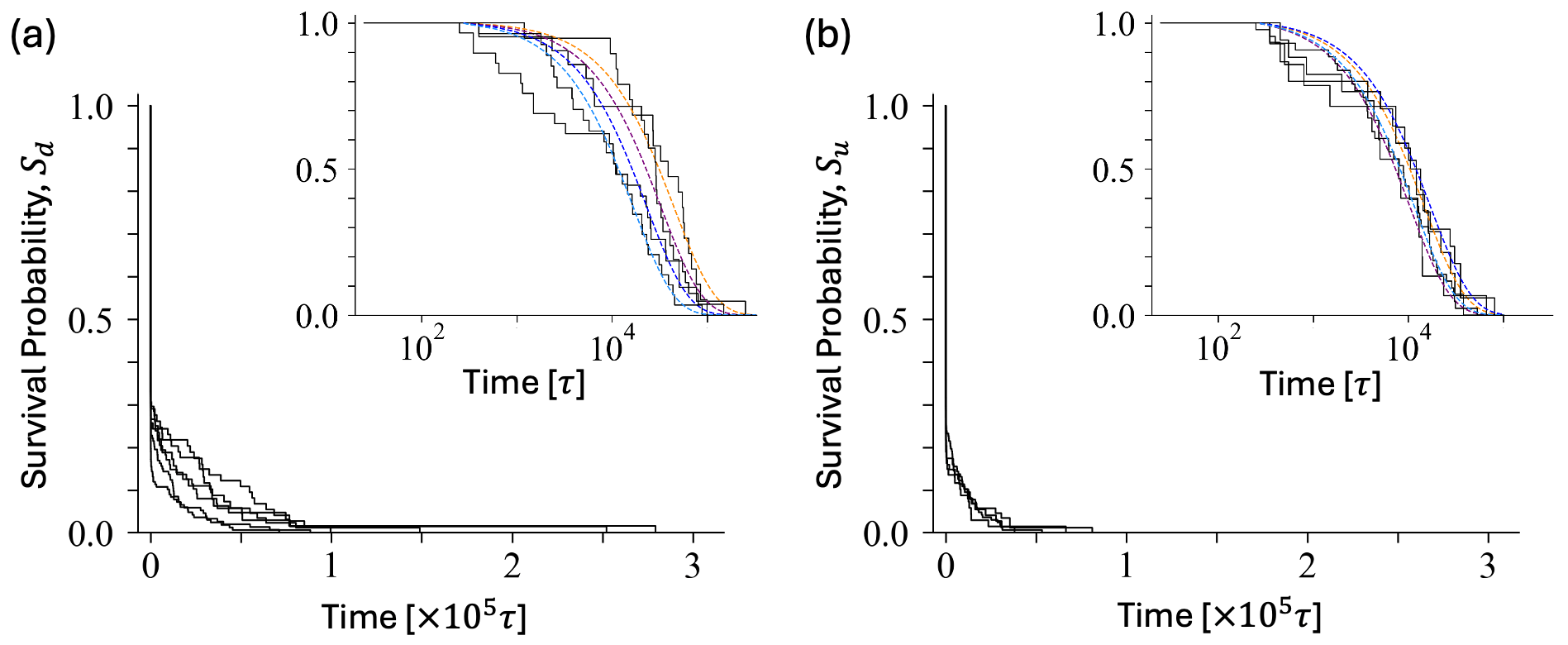

Supplement: Supplementary file 1 [file polymers-16-02928-s001.zip › Definitions/Fig4SP.png]

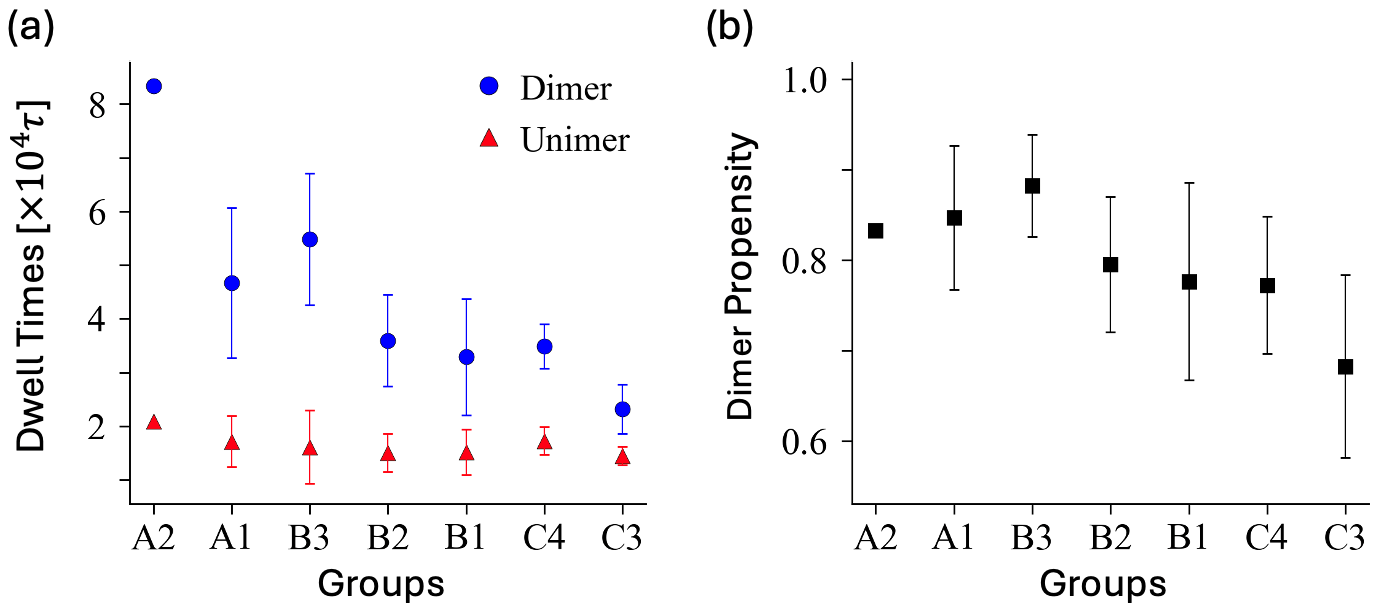

Supplement: Supplementary file 1 [file polymers-16-02928-s001.zip › Definitions/Fig5.png]

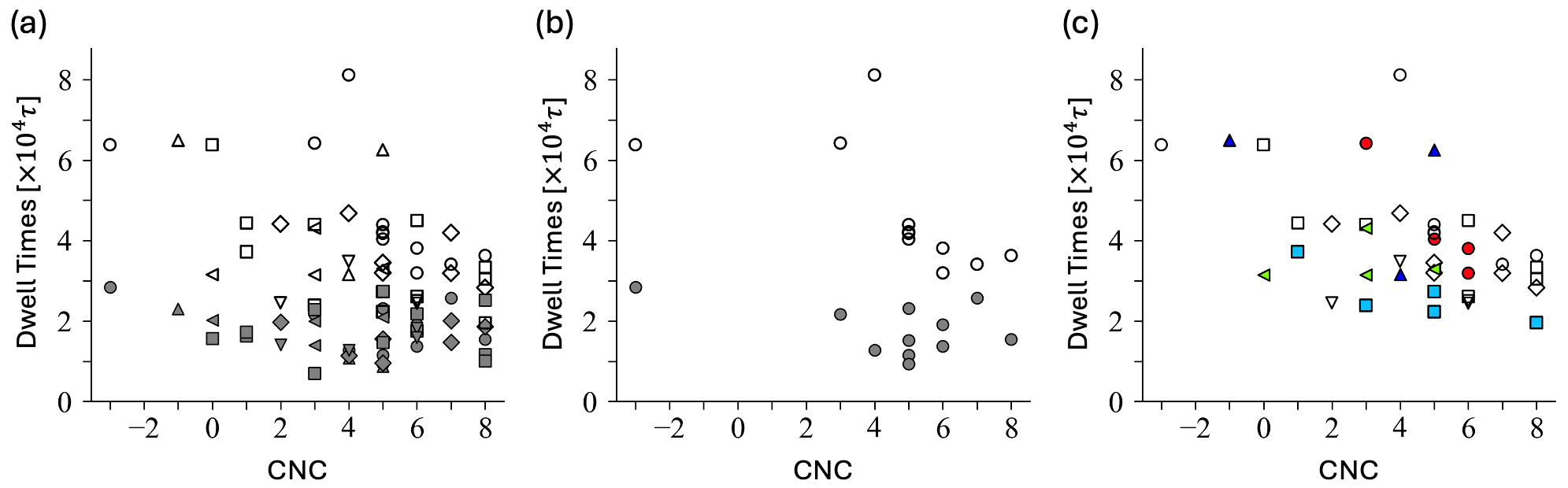

Supplement: Supplementary file 1 [file polymers-16-02928-s001.zip › Definitions/Fig6CNC.png]

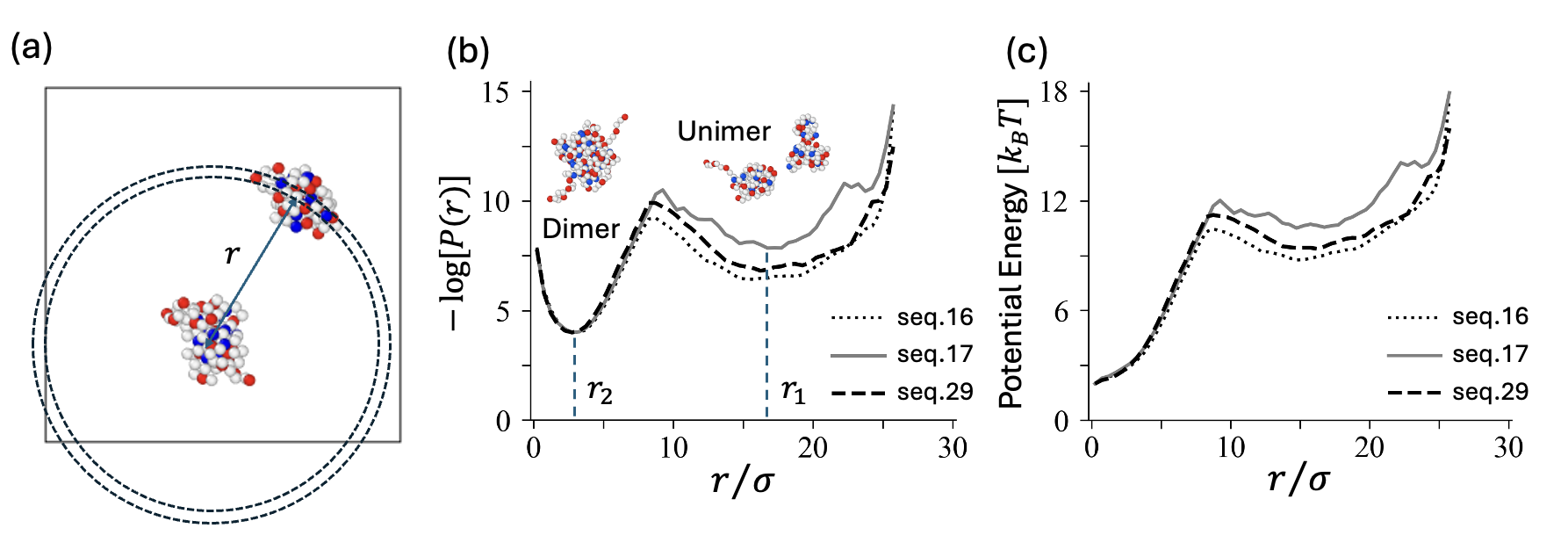

Supplement: Supplementary file 1 [file polymers-16-02928-s001.zip › Definitions/Fig7FE.png]

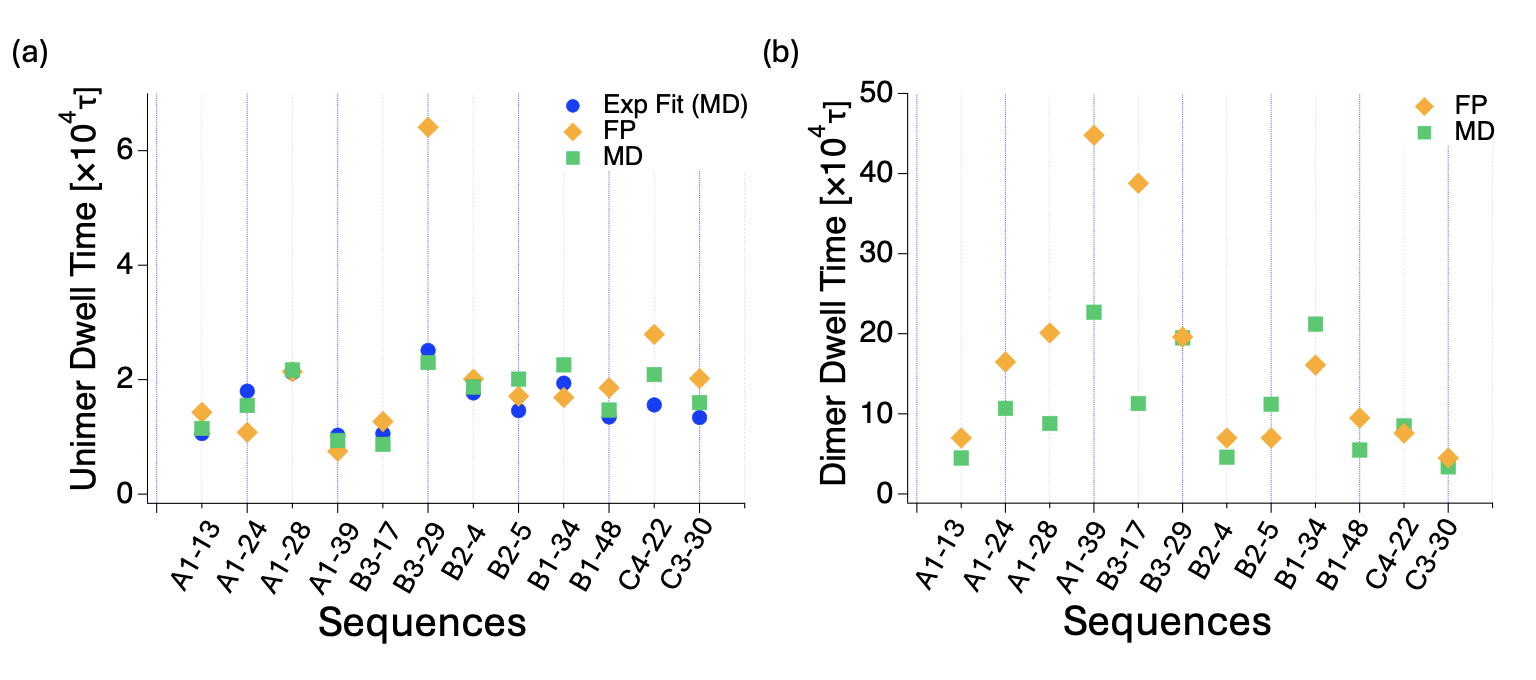

Supplement: Supplementary file 1 [file polymers-16-02928-s001.zip › Definitions/Fig8FP.png]

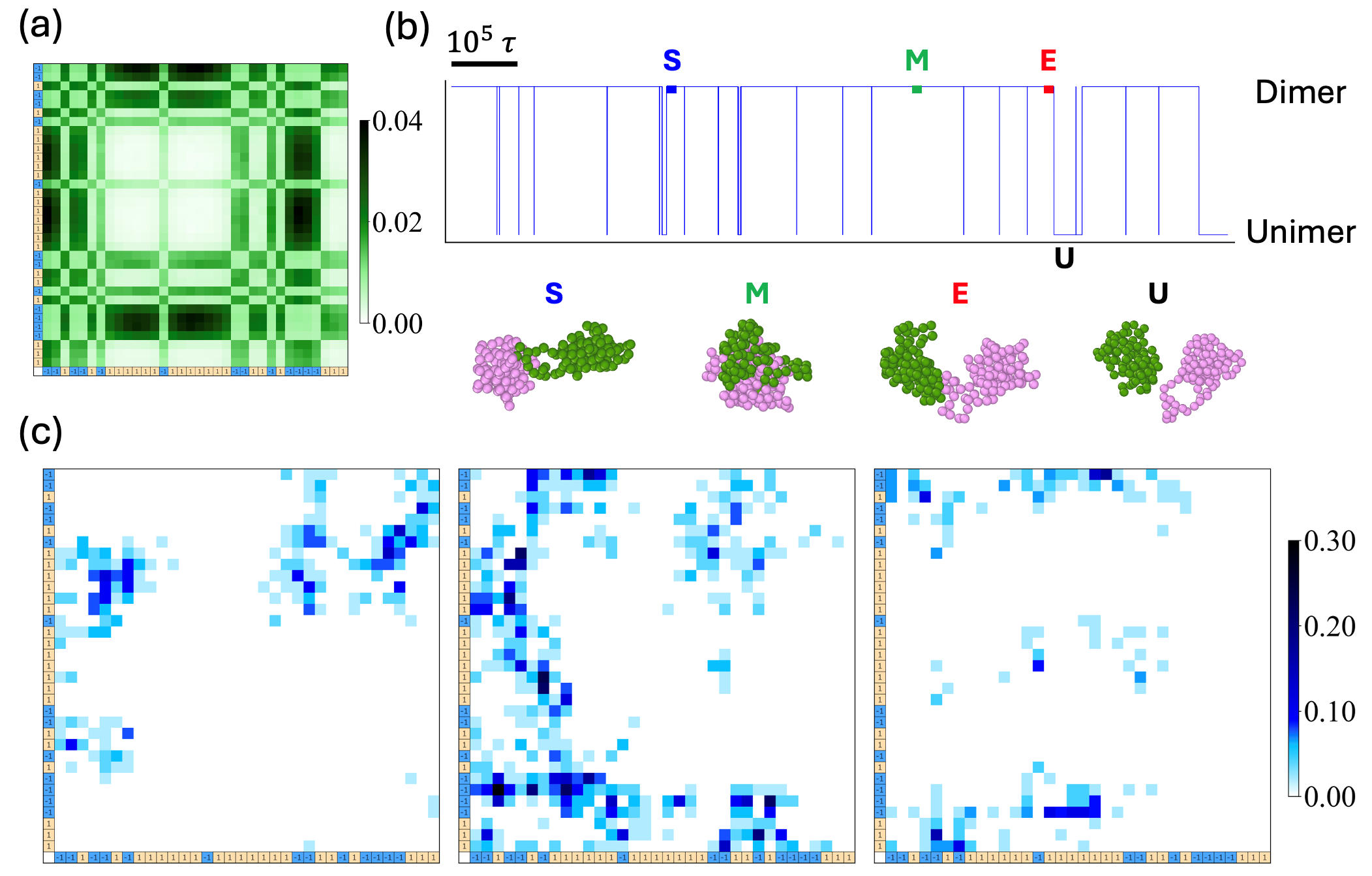

Supplement: Supplementary file 1 [file polymers-16-02928-s001.zip › Definitions/Fig9DCM.png]

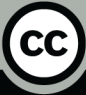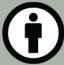

BY

Supplement: Supplementary file 1 [file polymers-16-02928-s001.zip › Definitions/logo-ccby-eps-converted-to.pdf]

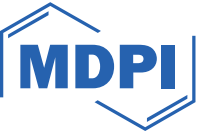

Supplement: Supplementary file 1 [file polymers-16-02928-s001.zip › Definitions/logo-mdpi-eps-converted-to.pdf]

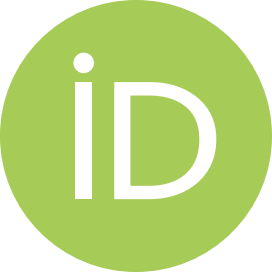

Supplement: Supplementary file 1 [file polymers-16-02928-s001.zip › Definitions/logo-orcid.pdf]

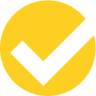

check for  
updates

Supplement: Supplementary file 1 [file polymers-16-02928-s001.zip › Definitions/logo-updates-eps-converted-to.pdf]

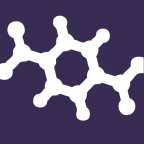

*polymers*

Supplement: Supplementary file 1 [file polymers-16-02928-s001.zip › Definitions/polymers-logo-eps-converted-to.pdf]

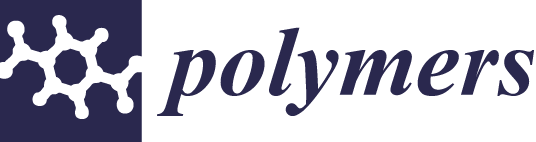

Supplement: Supplementary file 1 [file polymers-16-02928-s001.zip › Definitions/polymers-logo.png]

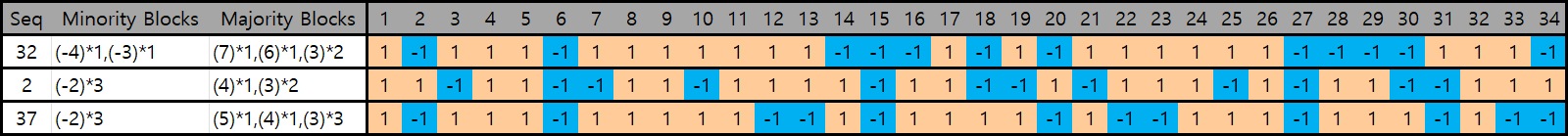

Supplement: Supplementary file 1 [file polymers-16-02928-s001.zip › Definitions/Q10_3seqs_0.png]

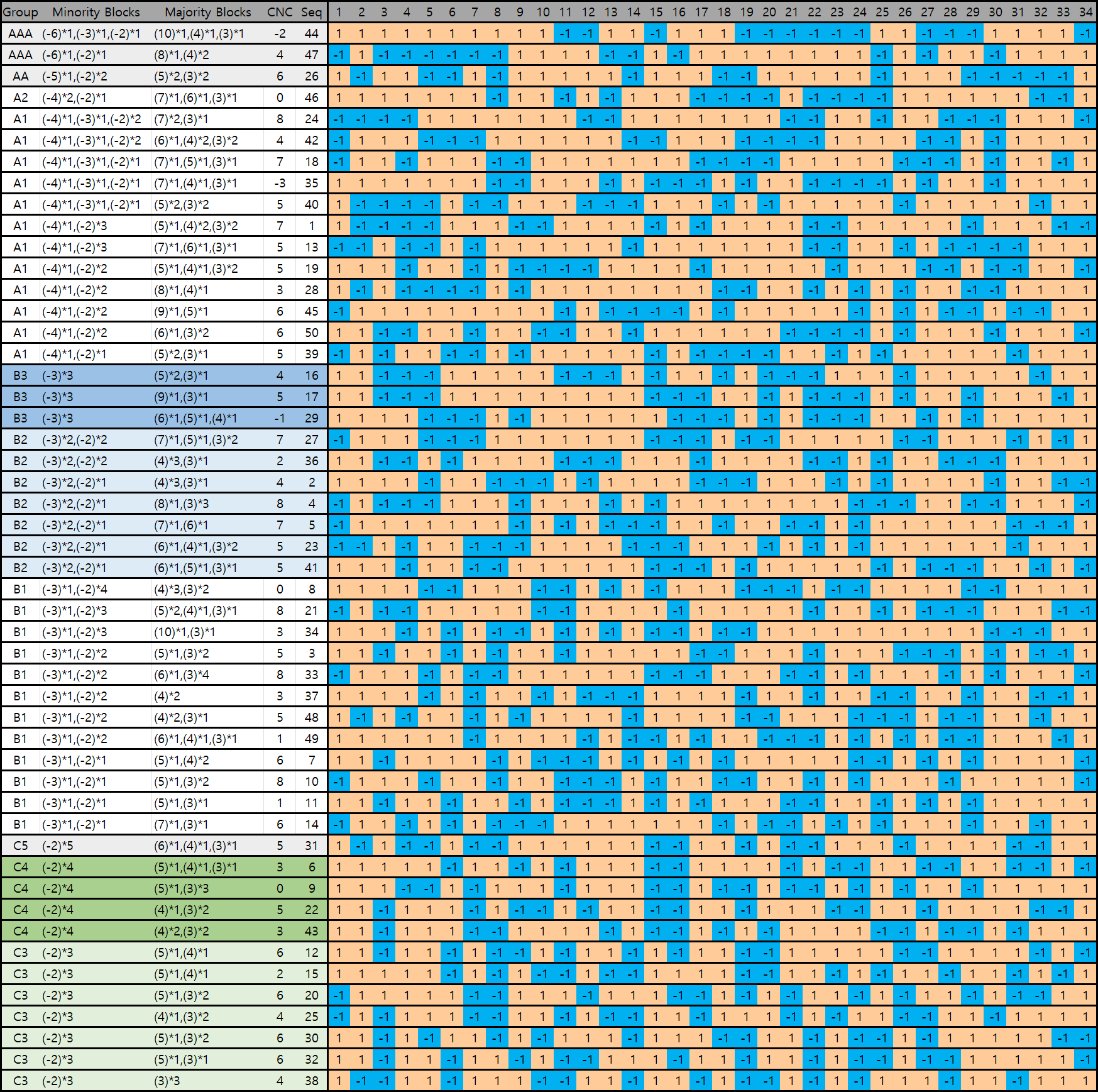

Supplement: Supplementary file 1 [file polymers-16-02928-s001.zip › Definitions/Table1.png]

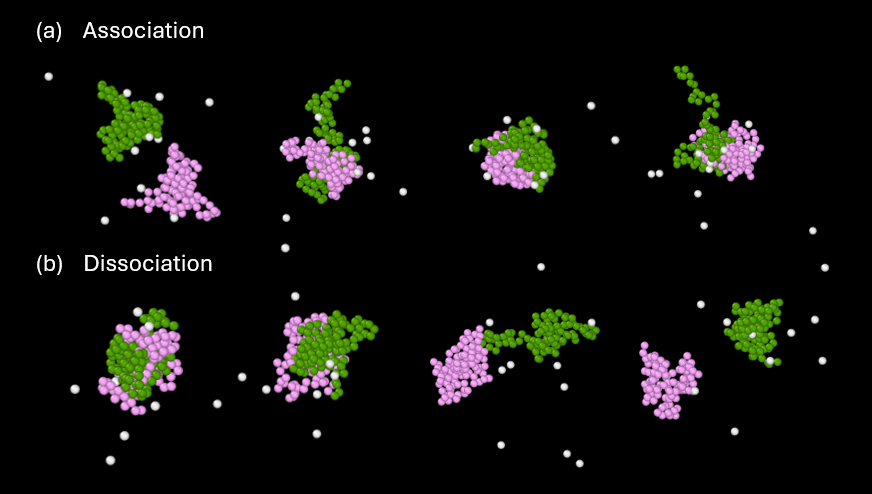

Supplement: Supplementary file 1 [file polymers-16-02928-s001.zip › FigureS1.png]

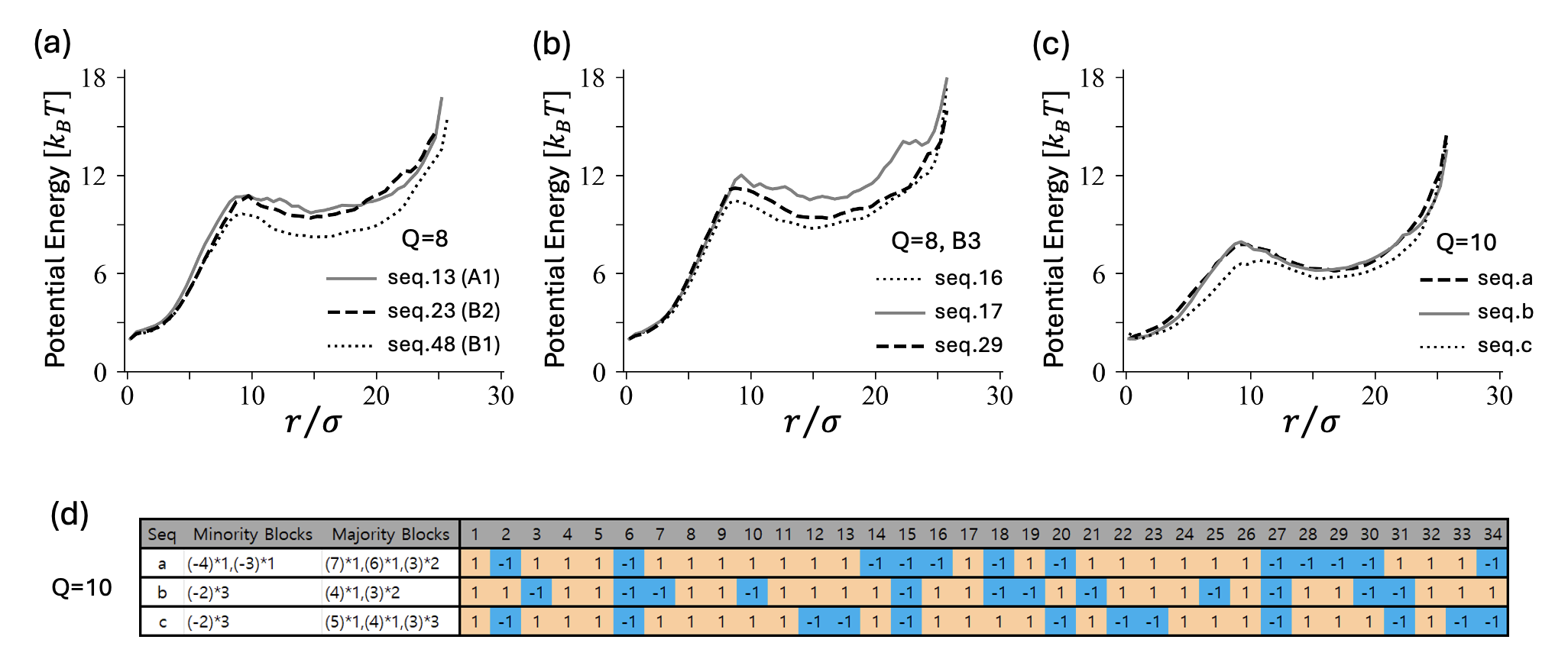

Supplement: Supplementary file 1 [file polymers-16-02928-s001.zip › FigureS2.png]
